# Work-Focused Versus Generic Internet-Based Interventions for Employees With Stress-Related Disorders: Randomized Controlled Trial

**DOI:** 10.2196/34446

**Published:** 2023-04-25

**Authors:** Robert Persson Asplund, Sofia Asplund, Helene von Buxhoeveden, Hanna Delby, Karin Eriksson, Maurits Svenning Gerhardsson, Joachim Palm, Thea Skyttberg, Julia Torstensson, Brjánn Ljótsson, Per Carlbring, Gerhard Andersson

**Affiliations:** 1 Department of Behavioural Sciences and Learning Linköping University Linköping Sweden; 2 Department of Psychology Uppsala University Uppsala Sweden; 3 Department of Clinical Neuroscience Karolinska Institutet Stockholm Sweden; 4 Department of Psychology Stockholm University Stockholm Sweden; 5 Department of Biomedical and Clinical Sciences Linköping University Linköping Sweden

**Keywords:** stress, burnout, exhaustion, work-focused, internet-based, intervention, sickness absence

## Abstract

**Background:**

In recent decades, stress-related disorders have received more attention, with an increasing prevalence, especially within the working population. The internet provides new options for broad dissemination, and a growing body of evidence suggests that web-based interventions for stress might be effective. However, few studies have examined the efficacy of interventions in clinical samples and work-related outcomes.

**Objective:**

The aim of this study was to evaluate the efficacy of an internet-based cognitive behavioral intervention for stress-related disorders integrating work-related aspects (work-focused and internet-based cognitive behavioral therapy [W-iCBT]), compared with a generic internet-based cognitive behavioral therapy (iCBT) group and a waitlist control (WLC) group.

**Methods:**

In this trial, 182 employees, mainly employed in the health care, IT, or educational sector, who fulfilled the criteria for a stress-related disorder, were randomized to a 10-week W-iCBT (n=61, 33.5%), generic iCBT (n=61, 33.5%), or WLC (n=60, 33%). Self-rated questionnaires on perceived stress, burnout, exhaustion, and other mental health– and work-related outcomes were administered before and after the treatment and at 6- and 12-month follow-ups.

**Results:**

Compared with the WLC group, participants of the W-iCBT and iCBT groups showed an equal and significant reduction in the primary outcome (Shirom-Melamed Burnout Questionnaire [SMBQ]) from pretreatment to posttreatment assessment (Cohen *d*=1.00 and 0.83, respectively) and at the 6-month follow-up (Cohen *d*=0.74 and 0.74, respectively). Significant moderate-to-large effect sizes were also found in the secondary health- and work-related outcomes. The W-iCBT was the only group that exhibited significant effects on work ability and short-term sickness absence. Short-term sickness absence was 445 days lower than the WLC group and 324 days lower than the iCBT intervention group. However, no significant differences were found in terms of work experience or long-term sick leave.

**Conclusions:**

The work-focused and generic iCBT interventions proved to be superior compared with the control condition in reducing chronic stress and several other mental health–related symptoms. Interestingly, effects on work ability and short-term sickness absence were only seen between the W-iCBT intervention and the WLC groups. These preliminary results are promising, indicating that treatments that include work aspects may have the potential to accelerate recovery and reduce short-term sickness absence because of stress-related disorders.

**Trial Registration:**

ClinicalTrials.gov NCT05240495; https://clinicaltrials.gov/ct2/show/NCT05240495 (retrospectively registered)

## Introduction

### Background

Work is an important part of life that contributes to both the health and well-being of many employees. However, in recent decades, stress has received more attention, with an increasing prevalence in the working population [1,2]. For example, every fourth employee within the European Union experiences stress during most of their working days [1]. Long-term exposure to stressors, such as job strain or interpersonal conflicts, without sufficient recovery, can lead to dysregulation of the allostatic system, which constitutes the fundamental feature in the development of chronic stress [3]. Chronic stress can lead to a wide range of disorders and clinical outcomes, including stress-related disorders [4-8]. According to the International Statistical Classification of Diseases and Related Health Problems, 10th Revision (ICD-10), stress-related disorders refer to a group of psychiatric disorders, including posttraumatic stress disorder, acute stress reaction, adjustment disorder, and other stress reactions after traumatic or stressful life events [2]. Nontraumatic stress disorders, such as adjustment disorders and other stress reactions, are usually triggered by identifiable stressors (eg, divorce or job loss). In this study, the term “stress-related disorders” refers to nontraumatic stress disorders. The major diagnostic systems, the ICD-10 and the Diagnostic and Statistical Manual of Mental Disorders, Fifth Edition (DSM-5), include sections regarding stress-related disorders. However, the DSM-5 and ICD-10 systems lack established terminology and criteria for stress-induced exhaustion. Consequently, the diagnosis of “exhaustion disorder” (ED) was introduced in the Swedish version of the ICD-10 in 2005 [9]. ED manifests as symptoms of extensive mental and physical fatigue, the lack of initiative and endurance, and prolonged recovery after mental or physical effort. Later international publications have suggested that ED is not an exclusively Swedish condition [10,11]. Few studies have been published regarding the prevalence of ED, but in a recent study, based on physician-based diagnosis in 3406 participants, 4.2% reported ED [12].

In addition to stress-related disorders and well-known health implications, such as coronary artery disease, lowered immune functioning, anxiety, depression, and insomnia [4-8], chronic stress has been associated with impaired work functioning and problems in work participation such as sickness absence (SA) and long-term sick leave [13-15]. Decreased work participation is problematic, as it has direct effects on people’s well-being and leads to immense costs for society [16]. For instance, the total estimated annual costs for work-related stress observed in 17 Organisation for Economic Co-operation and Development (OECD) countries are considerable, ranging from US $221 million to US $187 billion [16]. Given these rising costs, it is not surprising that many policy makers view stress as a major public health issue and seek advice on the types of interventions that may be effective [2].

During the last few decades, psychological interventions have been developed to increase individuals’ psychological resources and resilience to stress [17]. Evidence suggests that stress management interventions may be effective in reducing stress in the working population [17]. These results apply to controlled studies that target individual-level interventions and individuals with lower stress symptoms. However, considering organizational-level factors and clinical samples, interventions have been less successful [17,18].

Traditionally, psychological treatments for stress and common mental disorders (eg, depression or anxiety) have not explicitly focused on work-related aspects, such as organizational factors, (eg, demand, control, and support) or outcomes (eg, reducing SA). Recent evidence suggests that psychological interventions are slightly more effective than treatment-as-usual in reducing SA (small effect sizes). However, it remains uncertain what moderates these effects [19]. There are some indications that work-directed interventions, integrating work aspects (eg, increasing control and support) with individual psychological treatment, are effective in accelerating return to work (RTW) for those absent with common mental health problems [20-24]. In a quasi-experimental study by Lagervelt et al [20] comparing cognitive behavioral therapy (CBT) with work-focused CBT, full RTW occurred 65 days earlier and partial RTW occurred 12 days earlier in the work-focused CBT group. A substantial decrease in mental health problems was observed under both conditions. These results suggest that by integrating work-related aspects early into the treatment, problems with SA and long-term sick leave can be reduced.

Despite the well-documented efficacy of stress management interventions [17], and some promising results of work-focused interventions [20-25], the range of interventions is not proportionate to the needs of distressed employees [26]. This clarifies the need to further develop and evaluate work-directed interventions that are accessible to the working population.

Studies suggest that stress can be managed through internet- and computer-based interventions [27-30]. In a meta-analysis [27] including 26 controlled studies (n=4226), small to moderate effects were found on the outcomes of stress (Cohen *d*=0.43), depression (Cohen *d*=0.34), and anxiety (Cohen *d*=0.32). Subgroup analyses revealed that guided interventions (Cohen *d*=0.64) and interventions ranging between 5-8 weeks were more effective [27]. Studies also suggest that internet-based interventions for stress can have sustained effects on stress reduction [31-33], be cost-effective [34,35], and have positive effects on participants’ experiences of health and well-being in both work and private life [36]. However, previous internet-based cognitive behavioral therapy (iCBT) studies have focused on individuals with elevated stress, and few studies have evaluated the efficacy of iCBT in clinical samples such as employees with stress-related disorders. In addition, we found no previous studies evaluating the efficacy of internet-based and work-focused interventions for SA and RTW.

### Purpose of This Study

The aim of this study was to evaluate the efficacy of a work-focused iCBT (W-iCBT) intervention compared with generic iCBT and a waitlist control (WLC) group in a self-referred sample of employees with stress-related disorders. We hypothesized that W-iCBT and iCBT would be superior in reducing perceived stress, burnout, and exhaustion and improving recovery from work and quality of life compared with a WLC group. In secondary explorative analyses, we examined whether the W-iCBT group would differ from the iCBT and WLC groups in terms of important work-related outcomes, including work experience, work ability, SA, and long-term sick leave. We also hypothesized that the initially achieved changes would remain stable at the 12-month follow-up period.

## Methods

### Design

**Figure 1:**
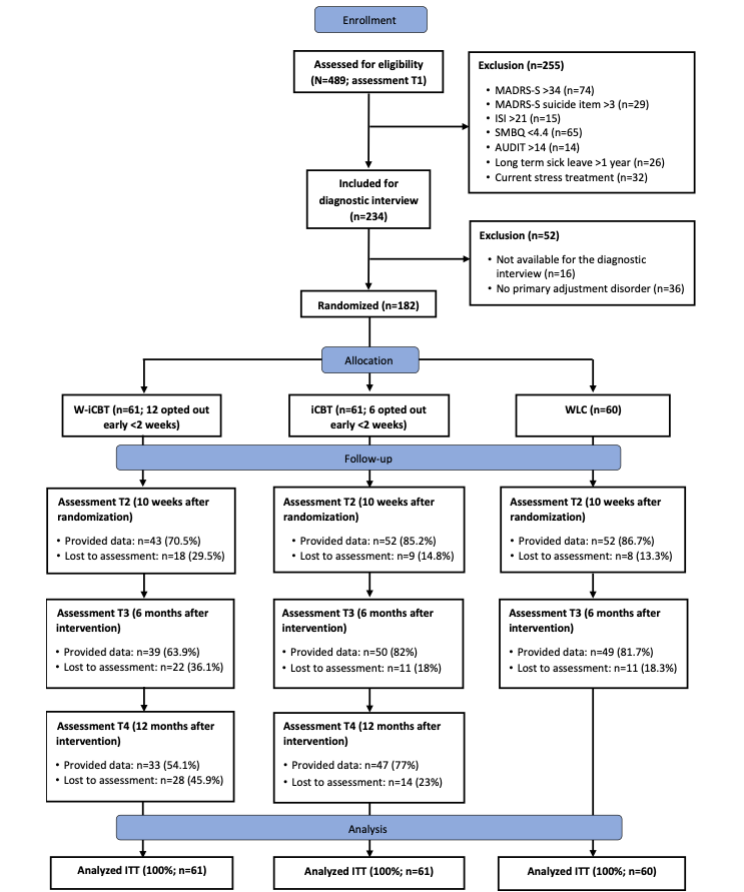
Flow of study participants. AUDIT: Alcohol Use Disorders Identification Test; iCBT: internet-based cognitive behavioral therapy; ISI: Insomnia Severity Index; ITT: intention to treat; MADRS-S: Montgomery-Åsberg Depression Rating Scale–Self-rating version; SMBQ: Shirom-Melamed Burnout Questionnaire; W-iCBT: work-focused and internet-based cognitive behavioral therapy; WLC: waitlist control.

This study was a 3-armed controlled superiority trial with 182 participants, and two internet-based interventions for stress-related disorders, namely, (1) iCBT (n=61, 33.5%) and (2) W-iCBT (n=61, 33.5%) that integrated work aspects early into the treatment, were compared against a WLC group (n=60, 33%). The study followed the CONSORT (Consolidated Standards of Reporting Trials) guidelines [37]. Estimates of sample size was based on previous controlled trials on iCBT for chronic stress [31], to detect an effect size of Cohen *d*=0.50 on the primary outcome of the Shirom-Melamed Burnout Questionnaire (SMBQ; *Measures* section) at posttreatment assessment, based on a power of 0.80 in a 2-tailed *t* test with .05 significant level. Self-reported outcome assessments were collected at pre- and posttreatment periods (10 weeks) and at 6- and 12-month follow-ups (Figure 1). Participants who met the study criteria and provided informed consent were randomly allocated by an independent researcher by using an internet-based random generator (Randomizer). The independent researcher received a list of anonymous identification numbers of all participants (n=182) and coaches (n=8). This procedure ensured that blinding was implemented during randomization. All participants, coaches, and the participants reporting benefits because of long-term sick leave at baseline were randomized in a 1:1:1 proportion.

### Ethics Approval

The Ethical Committee of Linköping University, Sweden, approved all procedures involved in this study (reference number 2016/11-31). The study was registered retrospectively due to delay at ClinicalTrials.gov (NCT05240495).

### Procedure

The study was conducted in a university setting, with researchers and the treatment platform hosted by the university. Participants were recruited from the public through advertisements, articles in regional and national newspapers, and labor organization magazines. Detailed information and application to the study were presented on the project’s home page. After initial registration using a personal email address, potential participants received an ID number and were asked to (1) provide written informed consent, (2) complete web-based screening questionnaires (*Measures* section), and (3) participate in a diagnostic interview via telephone. Diagnostic interviews were conducted by licensed psychologists or master’s-level psychology students under supervision. The master’s-level psychology students underwent diagnostic training, and all diagnostic interviews were reviewed by a licensed psychologist. Following the interviews, the included participants were randomized. Participants in the iCBT and W-iCBT groups received access to the programs immediately after randomization, and participants in the WLC group received access to the W-iCBT program after the 6-month follow-up period.

### Inclusion and Exclusion Criteria

The participants were employees who volunteered to participate in the trial. To be eligible for the study, they had to fulfill the criteria for an adjustment disorder described in the subdivision *F43 Reaction to severe stress and adjustment disorders* of the ICD-10 [38]. The diagnosis was established through telephone interviews using the Mini International Neuropsychiatric Interview [39], additional criteria from the ICD-10 [38], and national diagnostic guidelines regarding stress-related disorders [9].

In addition to an adjustment disorder, participants must (1) be aged ≥18 years; (2) have Swedish proficiency; (3) have access to a computer or tablet computer with internet access; (4) be currently employed; and (5) have scored ≥4.4 points on SMBQ, ≤34 points on the Montgomery-Åsberg Depression Scale–Self-rating version (MADRS-S), ≤21 points on the Insomnia Severity Index (ISI), and ≤14 points on the Alcohol Use Disorders Identification Test (AUDIT). Mild to moderate forms of DSM-5 axis-I diagnosis [40] were accepted as comorbid conditions, as long as these were considered secondary to the primary adjustment disorder. Participants on full- or part-time sick leave, for ≤1 year, were also included.

Participants were excluded from the study if they (1) were currently in treatment for a stress-related disorder; (2) were currently experiencing bipolar disorder, psychosis, posttraumatic stress disorder, eating disorder, substance abuse, severe forms of depression, anxiety disorder, or personality disorders; or (3) had suicidal ideation based on item 9 of the MADRS-S. Participants on medication (eg, antidepressants or sleep medication) were not excluded from the study but were requested to keep their medication constant during the study period. In total, 489 individuals were screened and 307 (62.8%) were excluded according to the inclusion and exclusion criteria specified above.

### Interventions

The generic iCBT program, represented in the iCBT and W-iCBT groups, was based on contemporary CBT techniques adapted for stress-related disorders and recovery from work training inspired by Hahn et al [41]. Both the iCBT and W-iCBT programs consisted of 10 modules distributed over 10 weeks, with modules lasting 60-120 minute per week (Table 1). The W-iCBT was integrated and distributed over each module and compared with generic iCBT, adding correspondingly 1 to 3 regular pages of text, worksheets, and homework assignments. Each module contained information, exercises, worksheets, images, examples, audio and video files, and homework exercises. All participants were requested to complete each module and homework assignment before they were able to continue. Delayed participants were able to catch up during the last module of the program. All participants had access to the treatment 1 year after the posttreatment assessment.

In the first module (introduction), the participants received information about the outline of the program and defined their individual treatment goals (eg, “I would like to be more assertive”). The first week also contained information and exercises on stress physiology; consequences of long-term stress, exhaustion, and burnout; and how to manage stressors (eg, workload, pace, and social support). During the second week (balance), the participants were introduced to different recovery techniques [41] and applied relaxation techniques [42]. These components recurred throughout the program. The third and fourth weeks included exercises related to behavioral activation [43], work-home balance, and value-based action skills derived from Acceptance Commitment Therapy [44]. Between weeks 5 and 10, participants were able to choose between different exposure-based exercises regarding assertiveness, perfectionism, procrastination, or worry. The final 3 weeks also comprised physical activity and time management. In addition, participants with insomnia could choose to focus on sleep management between weeks 5 and 9, including well-established CBT techniques such as sleep restriction, stimulus control, sleep hygiene, and cognitive interventions [45,46]. These sections were complementary and were used in parallel with the main program. In the last module, 10, a summary and prevention plan, including an evaluation of individual treatment goals and early signs of stress, was made.

The W-iCBT consisted of generic treatment for stress-related disorders (iCBT), plus components focusing on work and RTW. The W-iCBT aimed to facilitate RTW among those participants who were on sick leave and increase work functioning among those participants who were experiencing an adjustment disorder but not disabled from work. Work-focused CBT is built on the same conceptual framework as regular CBT. For example, CBT principles are used to change the appraisal of work stressors (eg, “it is ok although the task is not 100% complete or perfect”), change dysfunctional behavior (eg, working late close to bedtime and accepting more work despite heavy workload), or increase health-promoting behaviors (eg, recreational activities, assertive behavior, and RTW activities). The CBT principle of exposure has received special attention. Gradual exposure can help individuals develop more effective coping skills when dealing with work-related stressors (eg, assertiveness) and stimulate a gradual RTW setting for individuals on long-term sick leave [21].

In the first 4 modules, a work-related perspective on the symptoms of chronic stress was provided, and an analysis of the work situation was made (including job content, job conditions, work relations, and work satisfaction). In module 5, participants who were on sick leave made the first draft of a gradual (stepwise) RTW plan, and participants who were working made an elaborative work adjustment plan. Participants were encouraged to communicate their problem analysis, RTW, and work adjustment plans through dialogue with their employer (eg, manager and human resource professionals). These plans were continually updated by the participants throughout the remaining modules. In modules 6 to 10, participants were taught how to become involved in health-promoting behaviors at work, including (1) effort, appreciation, and reward; (2) control, responsibility, and autonomy; (3) social, instrumental, emotional, and informative support; and (4) recovery in the workplace. In the last module, 10, an integrated summary and prevention plan was drafted for both the work-focused and generic CBT programs.

**Table 1:** Cognitive behavioral therapy (CBT) and work-focused content.

Module	Name	CBT content	Work-focused content
1	Introduction	Psychoeducation about stress, establishing aims	Work content, tasks, demands, and workload
2	Balance	Recovery through psychological detachment, mindfulness, work and life balance, and values	Work conditions, employment type, status, and security
3	Stressful thoughts, feelings, and behaviors	Recovery through applied relaxation (step 1) stress diary, function relation between stress, appraisals, emotions, and behavior	Work relations, cooperation, leadership, organizational justice, feedback, social support, conflicts, harassment, and bullying
4	Recovery in everyday life	Work and life balance, applied relaxation (step 2) stress diary, coping with stressors, sleep management (step 1), and values	Job satisfaction, meaningfulness, and the importance of working for contentment
5	Challenges	Gradual exposure in a stress-related area, recovery through mastering new skills, applied relaxation (step 3), and sleep management (step 2)	Summary of the entire work situation, plan for workplace adjustments, and gradual return-to-work plan (only for participants on sick leave)
6	Challenges, continued	Continued exposure, values, applied relaxation (step 4), and sleep management (step 3)	Effort, appreciation and reward and continued plan for workplace adjustments and gradual return to work
7	Physical exercise	Recovery through physical exercise, applied relaxation (step 5), and sleep management (step 4)	Control, responsibility, and autonomy and continued plan for workplace adjustments and gradual return to work
8	Planning	Time management, values, applied relaxation (step 6), and sleep management (step 5)	Social, instrumental, emotional, and informative support and continued plan for workplace adjustments and gradual return to work
9	Cognitive functioning	Stress, burnout, and cognitive functioning; how to manage distractions and temporary memory problems; and applied relaxation (step 7)	Recovery in the workplace and continued plan for workplace adjustments and gradual return to work
10	Action and relapse prevention plan	Evaluation of training, early warning signs, values, summary, and prevention plan	Summary and prevention plan

### Support

Every week, participants in both interventions (W-iCBT and iCBT) received personalized written messages from a coach with feedback on the exercises. For the participants in the W-iCBT group, guidance was given on the CBT and work-focused modules simultaneously. The coaches, 8 in total, were master’s-level psychology students who were specifically trained to provide feedback according to a standardized manual. The feedback aimed to provide support and encouragement and to monitor homework assignments and adherence to the intervention. Treatment-as-usual was not only prohibited but also not encouraged during the trial. The coaches were requested to limit their support to 1 message and a maximum of 15 minutes of correspondence per week with 1 participant.

### Primary Outcome Measure *Shirom Melamed Burnout Questionnaire*

The Shirom Melamed Burnout Questionnaire (SMBQ) [47,48] is a 22-item scale (graded 1-7) used to assess different aspects of chronic stress and burnout (physical fatigue, cognitive weariness, tension, and listlessness). This scale correlates signiﬁcantly with other well-established questionnaires measuring burnout, for example, the Maslach Burnout Inventory [48]. The SMBQ has exhibited good internal consistency with a Cronbach α of .92 [49] and in this study, indicated by an α of .84.

### Secondary Outcome Measures

#### Perceived Stress Scale

Perceived stress was measured using the 10-item version of the Perceived Stress Scale (PSS-10), translated into Swedish [50,51]. The PSS-10 is designed to measure the degree to which situations in one’s life are appraised as stressful. The Swedish version of the PSS-10 has an internal consistency reliability (Cronbach α) of .82 and in the present sample, of .77.

#### Karolinska Exhaustion Disorder Scale

Karolinska Exhaustion Disorder Scale (KEDS-9) is a 9-item questionnaire measuring symptoms of chronic stress, fatigue, and exhaustion [52]. The instrument is answered on a 7-point scale, with a scale range of 0 to 54. A cutoff score of 19 was shown to discriminate between healthy participants and patients with chronic stress and exhaustion [52]. The KEDS-9 has satisfactory reliability, with a Cronbach α of .94 [52] and correspondingly .74 for this trial.

#### Montgomery Åsberg Depression Rating Scale

We used the Montgomery Åsberg Depression Rating Scale self-assessment (MADRS-S) [53] to measure symptoms of depression. The MADRS-S consists of 9 items measuring different symptoms of depression, and each symptom is rated on a 6-point scale. The instrument has good reliability [54] indicated by a Cronbach α of .75 in this study sample. In a comparative study [55], the MADRS-S correlated highly (*r*=.87) with the Beck Depression Inventory [56], indicating acceptable convergent validity.

#### Generalized Anxiety Disorder Scale

The Generalized Anxiety Disorder 7-item Scale (GAD-7) is an instrument used to assess excessive worry and generalized anxiety disorder. The GAD-7 has good internal consistency reliability (α=.83); test-retest reliability (*r*=.83); as well as criterion, construct, factorial, and procedural validity [57]. A Cronbach α of .79 was obtained in this study. A cutoff score of 10 has been suggested to discriminate between healthy participants and patients with generalized anxiety disorders.

#### Insomnia Severity Index

The Insomnia Severity Index (ISI) is a 7-item self-report questionnaire that measures individuals’ perceptions of their insomnia and the severity of problems with delayed sleep onset, sleep maintenance, and early morning awakenings [58]. The ISI exhibits adequate internal consistency measures (α=.74) and is a sensitive measure for detecting changes in perceived sleep difficulties [58]. In this study sample, the Cronbach α was .86. It has previously been validated as an internet-based measure [59].

#### Alcohol Use Disorders Identification Test

The Alcohol Use Disorders Identification Test (AUDIT) [60] was used to assess potential alcohol dependence or abuse. In a study of the psychometric properties of the Swedish version of the AUDIT, both internal and test-retest reliabilities were satisfactory [61]. In this study, the Cronbach α was .65. A cutoff of <14 points on the AUDIT indicates a risk of alcohol overconsumption [62].

#### Work Experience Measurement Scale

The Work Experience Measurement Scale (WEMS) is an instrument measuring the experience of work from a health resource perspective [63]. The WEMS consists of 32 items that measure job satisfaction in 5 different domains (supportive work conditions, internal work experience, autonomy, time experience, management, and process of change) on a 6-point scale. The Cronbach α for the WEMS was reported to be in the interval of .85 to .96 [63] and .94 in the present sample.

#### Work Ability Index

The Work Ability Index (WAI) is an instrument used to assess health status and work ability among employees [64-66]. The WAI comprises different scales, with scores ranging from 7 to 49. Studies [64] have suggested that 7 to 27 points indicate poor work ability; 28 to 36 points moderate ability; 37 to 43 points good ability; and 44 to 49 points indicate excellent work ability. Analyses of reliability indicate satisfactory internal consistency, with α levels ranging from .79 to .80 [67,68]. In this study, the α level was .66.

#### Sheehan Disability Scale

The Sheehan Disability Scale (SDS) measures quality of life and everyday function in 3 domains: work ability, social life, and family life [69,70]. The instrument is answered on a 10-point visual analog scale, with a scale range of 0 to 30. The SDS has satisfactory internal consistency reliability (α=.89) and test-retest reliability (*r*=0.73). The α level was .66 in this study sample [69,70].

#### Recovery Experiences Questionnaire

The 16-item Recovery Experience Questionnaire (REQ) includes four factors, representing four different recovery experiences: (1) psychological detachment, (2) relaxation, (3) mastery, and (4) control [71]. The questionnaire is answered on a 5-point Likert scale and has been validated in a Swedish population, showing excellent internal consistency (α=.92) [72]. In this study, the α level was .86.

#### Sickness Absence And Long-term Sick Leave

Absence from work was measured according to the Trimbos and Institute of Medical Technology Assessment Cost Questionnaire for Psychiatry (TiC-P) [73]. The TiC-P has been used in several studies for economic evaluation of health care consumption and productivity loss in mental health [73]. Sickness Absence (SA) was conceptualized as the self-rated number of days absent from work during the past 3 months while being physically or mentally ill. Long-term sick leave was operationalized as >15 days on sick leave and based on data from the Swedish Social Insurance Agency on the number of net days on sickness benefit between the pretreatment and 6-month follow-up assessments. In Sweden, sickness benefits from the Swedish Social Insurance Agency are due from day 15 on sick leave. Thus, absence during the first 14 days of illness was not included in the analysis of this outcome.

#### Intervention Utility And Satisfaction

The participants were asked to rate their utility and satisfaction after each module on a 5-point scale (1=low utility/satisfaction to 5=high utility/satisfaction).

#### Intervention Support

Intervention support was operationalized and assessed as the number of minutes of support per week between the coaches and the participants during the intervention.

### Statistical Analysis

All analyses followed the CONSORT statement for randomized controlled trials [37]. Statistical analyses were conducted following the intention-to-treat (ITT) principle using SPSS (version 26; IBM Corp). We used the multiple imputation procedure to impute missing sum scores for participants who did not complete the posttreatment and 6- or 12-month follow-up assessments. Multiple imputation is considered a conservative approach for analyzing incomplete data sets, as it takes into account the uncertainty because of missing information [74]. We used all available data from the pretreatment, posttreatment, and 6-month follow-up assessments, as well as age, gender, and educational level as predictors. Means, SDs, and SEs of the effect sizes were pooled from 5 sets of imputations. The effects of group on primary and secondary outcome measures of the ITT and completers-only data sets were analyzed using repeated measures ANOVAs with time (pretreatment, posttreatment, and 6-month follow-up period) as a within-subject factor. Pooled *F* values were calculated using RStudio (RStudio Inc). Cohen *d* was reported for between-group effect sizes and the corresponding 95% CI. Internal consistency reliability for the primary and secondary outcomes was analyzed using Cronbach α. Outcomes at baseline and demographic variables between complete and missing data were analyzed using 2-tailed *t* test and *χ*^2^ test. The ITT principle was applied to the analysis of SA. The analysis of long-term sick leave was based on complete registry data with no attrition. Both SA and long-term sick leave were analyzed using the Kruskal-Wallis nonparametric test, recommended for the comparison of ≥3 samples. To evaluate clinically significant changes, we used the guidelines by Jacobson and Truax [75]. Clinically significant changes were based on ITT analysis. To meet the criteria for clinically significant change in the primary outcome SMBQ, participants had to demonstrate a reliable change of 0.69 and score less than the cutoff of 4.4, following a recent study in a clinical sample [76]. We performed a clinically significant change analysis using the KEDS. On the KEDS, participants had to demonstrate a reliable change of 8.72 and score under the cutoff of 19 [52].

## Results

### Participants

Figure 1 shows the flow of participants, including those who were excluded. After screening 489 individuals, 307 participants were excluded, most (n=197) because of high or low scores on one or several of the outcome measures. A total of 182 participants were randomized to the W-iCBT (61/182, 33.5%), iCBT (61/182, 33.5%), or WLC (60/182, 33%) groups.

### Missing Data

Baseline data were available for all participants. Overall, the study attrition rate was moderate: 19.2% (35/182) at posttreatment period (W-iCBT: 18/35, 51%; iCBT: 9/35, 26%; and WLC: 8/35, 23%), 24.2% (44/182) at the 6 months follow-up (W-iCBT: 22/44, 50%; iCBT: 11/44, 25%; and WLC: 11/44, 25%), and 34.4% (42/122) at the 12-month follow-up questionnaires (W-iCBT: 28/42, 67% and iCBT: 14/42, 33%). The analysis found no significant differences (*χ*^2^_3_=.0645, n=182, *P*=.37) in the study attrition rate between any group or time point. No significant differences were found between demographic variables (presented in Table 2) or complete and missing data on the baseline outcome (Table 3).

**Table 2:** Baseline characteristics.

Characteristics			All participants (N=182)	W-iCBT^a^ (n=61)	iCBT^b^ (n=61)	WLC^c^ (n=60)
**Sociodemographics**						
	Age (years), mean (SD)		46.4 (8.6)	46.8 (8.6)	45.8 (9.0)	46.5 (8.3)
	Gender, woman, n (%)		147 (80.8)	46 (75.4)	51 (83.6)	50 (83.3)
	Married or in a relationship, n (%)		148 (81.3)	52 (85.2)	44 (72.1)	52 (86.7)
**Education, n (%)**						
	Low		0 (0)	0 (0)	0 (0)	0 (0)
	Middle		15 (8.2)	2 (3.3)	4 (6.6)	9 (15)
	High		167 (91.8)	59 (96.7)	57 (93.4)	51 (85)
**Working characteristics**						
	Full-time work		153 (84.1)	50 (82)	55 (90.2)	48 (80)
	Part-time work		29 (15.9)	11 (18)	6 (9.8)	12 (20)
	**Disability level (sickness benefit), n (%)**		51 (28)	17 (27.9)	17 (27.9)	17 (28.3)
		100%	23 (12.6)	8 (13.1)	6 (9.8)	9 (15)
		75%	9 (5)	3 (4.9)	4 (6.6)	2 (3.3)
		50%	17 (9.3)	6 (9.8)	6 (9.8)	5 (8.3)
		25%	2 (1.1)	0 (0)	1 (1.6)	1 (1.7)
	Hours of overtime, per week, mean (SD)		2.7 (3.3)	2.9 (3.1)	2.5 (3.3)	2.8 (3.4)
	Work experience in years, mean (SD)		6.0 (6.3)	6.6 (6.7)	4.8 (4.3)	6.7 (7.4)
**Working sectors, n (%)**						
	Social or health		60 (33)	20 (32.8)	17 (27.9)	23 (38.3)
	Education or research		52 (28.6)	18 (29.5)	17 (27.9)	17 (28.3)
	Communication or IT		18 (9.9)	8 (13.1)	7 (11.5)	3 (5)
	Law, economy, or technology		8 (4.3)	6 (9.8)	1 (1.6)	1 (1.7)
	Others		44 (24.2)	9 (14.8)	19 (31.1)	16 (26.7)
Mean income in US $ per year (SEK 1=US $ 0.095), mean (SD)			43,985 (16,309)	43,060 (14,887)	45,802 (20,005)	42,945 (13,436)
**Experience, n (%)**						
	Previous treatment		101 (55.5)	36 (59)	33 (54.1)	32 (53.3)
	First-time help seeker		66 (36.3)	21 (34.4)	23 (37.7)	22 (36.7)
**Primary disorders, n (%)**						
	F43.2 Adjustment disorder		29 (15.9)	6 (9.8)	13 (21.3)	10 (16.7)
	F43.8 Exhaustion disorder		140 (76.9)	48 (78.7)	46 (75.4)	46 (76.7)
	F43.9 Reaction to severe stress, unspecified		13 (7.2)	7 (11.5)	2 (3.3)	4 (6.6)
**Secondary disorders, n (%)**						
	F51.0 Nonorganic insomnia		121 (66.7)	44 (72.1)	40 (65.6)	37 (61.7)
	F41.1 Generalized anxiety disorder		50 (27.6)	25 (41)	10 (16.4)	15 (25)
	F32.x Depressive episode		61 (33.5)	15 (24.6)	15 (24.6)	31 (51.7)
	F33.x Recurrent depressive disorder		38 (20.9)	11 (18)	9 (14.8)	18 (30)
	F41.0 Panic disorder		21 (11.5)	5 (8.2)	6 (9.8)	10 (16.7)
	F40.1 Social phobia		15 (8.2)	11 (18)	2 (3.3)	2 (3.3)
	Noncomorbid		64 (35.2)	21 (34.4)	24 (39.3)	19 (31.7)

^a^W-iCBT: work-focused and internet-based cognitive behavioral therapy.

^b^iCBT: internet-based cognitive behavioral therapy.

^c^WLC: waitlist control.

**Table 3:** Means and SDs for the intention-to-treat sample (work-focused and internet-based cognitive behavioral therapy [W-iCBT]: n=61; internet-based cognitive behavioral therapy [iCBT]: n=61; and waitlist control [WLC]: n=60) at pretreatment (T1), posttreatment (T2), 6-month follow-up (T3), and 12-month follow-up (T4) time points.

Outcome						T1, mean (SD)			T2, mean (SD)			T3, mean (SD)			T4, mean (SD)
**Primary outcome**															
	**Burnout (1-7)^a^**														
		W-iCBT			5.09 (0.69)			3.64 (1.01)			3.58 (1.16)			3.25 (1.23)	
		iCBT			5.08 (0.65)			3.76 (1.11)			3.59 (1.15)			3.24 (1.23)	
		WLC			5.18 (0.56)			4.61 (0.94)			4.39 (1.02)			N/A^b^	
		**Emotional fatigue**													
			W-iCBT	5.05 (0.77)			3.53 (1.16)			3.36 (1.15)			3.25 (1.33)		
			iCBT	5.02 (0.87)			3.58 (1.23)			3.4 (1.2)			3.17 (1.42)		
			WLC	5.13 (0.74)			4.41 (1.1)			4.27 (1.16)			N/A		
		**Cognitive weariness**													
			W-iCBT	5.07 (0.95)			4.02 (1.13)			4.02 (1.19)			3.7 (1.27)		
			iCBT	5.04 (0.8)			4.05 (1.1)			3.89 (1.14)			3.62 (1.32)		
			WLC	5.27 (0.76)			5.02 (1.05)			4.83 (1.1)			N/A		
		**Tension**													
			W-iCBT	5.16 (0.88)			3.71 (1.15)			3.69 (1.23)			3.51 (1.29)		
			iCBT	5.12 (0.84)			3.82 (1.2)			3.77 (1.43)			3.26 (1.43)		
			WLC	5.23 (0.97)			4.71 (1.24)			4.39 (1.38)			N/A		
		**Listlessness**													
			W-iCBT	5.11 (1.11)			3.41 (1.21)			3.58 (1.4)			3.21 (1.37)		
			iCBT	5.16 (1.15)			3.57 (1.3)			3.52 (1.53)			3.26 (1.49)		
			WLC	5.15 (1.01)			4.49 (1.23)			4.21 (1.25)			N/A		
**Health related**															
	**Perceived stress (0-40)^c^**														
		W-iCBT			24.28 (4.98)			16.9 (6.54)			16.32 (6.31)			15.43 (6.96)	
		iCBT			24.21 (5.35)			17.72 (5.5)			16.93 (6.05)			14.97 (7.21)	
		WLC			24.73 (4.14)			21.87 (5.03)			20.79 (5.3)			N/A	
	**Exhaustion (0-54)^d^**														
		W-iCBT			29.8 (7.56)			19.46 (9.02)			19.39 (9.02)			16.52 (8.29)	
		iCBT			28.44 (6.66)			19.71 (7.88)			18.85 (8.08)			16.93 (8.42)	
		WLC			29.97 (4.86)			26.1 (6.88)			23.7 (8.2)			N/A	
	**Depression (0-54)^e^**														
		W-iCBT			19.43 (6.15)			11.98 (6.2)			11.62 (6.42)			9.62 (6.45)	
		iCBT			18.84 (5.76)			11.98 (6.55)			11.43 (6.76)			10.37 (7.2)	
		WLC			19.77 (6.09)			17.31 (6.55)			14.97 (7.22)			N/A	
	**Anxiety (0-21)^f^**														
		W-iCBT			9.52 (4.9)			5.34 (3.59)			4.6 (3.2)			3.8 (2.83)	
		iCBT			9.7 (4.12)			5.25 (3.6)			5.39 (3.93)			4.31 (3.89)	
		WLC			9.73 (4.06)			8.09 (4.26)			6.03 (3.61)			N/A	
	**Insomnia (0-28)^g^**														
		W-iCBT			13.75 (5.63)			8.16 (5.08)			8.52 (5.13)			7.3 (5.24)	
		iCBT			12.97 (6.03)			7.72 (5.46)			7.96 (6.09)			6.73 (5.15)	
		WLC			13.4 (6)			12.95 (5.95)			11.3 (5)			N/A	
	**Alcohol (0-40)^h^**														
		W-iCBT			2.77 (2.11)			2.73 (2.36)			N/A			N/A	
		iCBT			3.18 (2.55)			2.96 (2.14)			N/A			N/A	
		WLC			3.23 (2.4)			3.03 (2.33)			N/A			N/A	
	**Quality of life (0-30)^i^**														
		W-iCBT			18.66 (4.88)			13.27 (7.33)			11.71 (7.47)			10.48 (7.45)	
		iCBT			17.66 (6.07)			13.93 (6.73)			11.46 (6.54)			10.12 (6.81)	
		WLC			19.18 (3.95)			16.51 (5.44)			15.8 (6.5)			N/A	
**Work related**															
	**Work experience (32-192)^j^**														
		W-iCBT			120.02 (25.28)			126.44 (29.24)			129.22 (29.58)			130.56 (33.65)	
		iCBT			119.08 (26.13)			127.66 (29.06)			131.49 (27.12)			134.73 (31.66)	
		WLC			119.08 (28.75)			121.46 (32.91)			120.54 (31.89)			N/A	
	**Work ability (7-49)^k^**														
		W-iCBT			30.6 (5.76)			33.59 (6.85)			34.9 (7.6)			35.63 (9.55)	
		iCBT			30.39 (6.74)			32.45 (7.29)			33.92 (7.08)			36.66 (7.26)	
		WLC			29.98 (5.62)			30.65 (6.17)			32.65 (6.6)			N/A	
	**Recovery (16-80)^l^**														
		W-iCBT			45.21 (8.31)			52.35 (10.12)			52.53 (10.49)			53.57 (11.55)	
		iCBT			44.98 (8.82)			52.22 (9.96)			51.37 (10.88)			53.17 (10.7)	
		WLC			43.12 (10.64)			43.73 (9.27)			45.43 (11.62)			N/A	
		**Psychological detachment (4-20)**													
			W-iCBT	10.9 (3.09)			13.46 (2.97)			13.24 (3.07)			14.13 (3.25)		
			iCBT	10.54 (3.35)			12.99 (3.28)			13.09 (3.47)			13.49 (3.35)		
			WLC	10.2 (3.18)			10.86 (2.65)			11.54 (3.68)			N/A		
		**Relaxation (4-20)**													
			W-iCBT	11.82 (2.39)			14.17 (2.93)			13.73 (2.66)			14.11 (3.02)		
			iCBT	11.48 (2.85)			13.76 (2.98)			13.63 (2.98)			13.81 (3.17)		
			WLC	11.33 (2.87)			11.4 (2.81)			11.96 (3.28)			N/A		
		**Mastery (4-20)**													
			W-iCBT	9.2 (3.03)			10.74 (3.62)			11.02 (3.68)			10.94 (3.86)		
			iCBT	9.46 (3.14)			10.5 (3.46)			10.53 (3.71)			11.08 (3.74)		
			WLC	8.7 (3.51)			8.72 (3.32)			8.8 (3.47)			N/A		
		**Control (4-20)**													
			W-iCBT	13.3 (3.27)			14.32 (3.48)			14.03 (3.38)			14.8 (3.31)		
			iCBT	13.51 (3.66)			14.74 (3.09)			14.41 (3.34)			15.09 (3.13)		
			WLC	12.88 (4.05)			12.48 (3.54)			12.74 (3.67)			N/A		

^a^SMBQ: Shirom-Melamed Burnout Questionnaire.

^b^N/A: not applicable.

^c^PSS-10: Perceived Stress Scale.

^d^KEDS: Karolinska Exhaustion Disorder Scale.

^e^MADRS-S: Montgomery-Åsberg Depression Rating Scale–Self-rating version.

^f^GAD-7: Generalized Anxiety Disorder 7-item scale.

^g^ISI: Insomnia Severity Index.

^h^AUDIT: Alcohol Use Disorders Identification Test.

^i^SDS: Sheehan Disability Scale.

^j^WEMS: Work Experience Measurement Scale.

^k^WAI: Work Ability Index.

^l^REQ: Recovery Experience Questionnaire.

### Baseline Characteristics

Baseline characteristics of the study participants are presented in Table 2. The sample comprised 182 employees, most participants identified as women (n=147, 80.8%), with an average age of 46.4 (SD 8.6) years. A majority, that is, 112 (61.6%) participants were working in the social, health care, or educational sector. In total, 51 (28%) participants were on sick leave. The average participants were working full time (n=153, 84.1%), made 2.7 (SD 3.3) hours of overtime per week, had 6.0 (SD 6.3) years of work experience, and fulfilled the ICD-10 diagnosis: F43.8 ED (n=140, 76.9%) and F51.0 nonorganic insomnia (n=127, 66.7%).

### Adherence

On average, participants in the W-iCBT and iCBT groups completed 8.86 (SD 1.96) modules and 8.69 (SD 1.86) modules, respectively, which equals to 88.6% and 86.9% of each intervention. A significant proportion (*χ*^2^_30_=126.4, n=122, *P≤*.001) of participants, dropped out early (<2 weeks) in the W-iCBT group (15/61, 24.6%) compared with the iCBT group (6/61, 9.8%). The main reason for dropping out was a lack of time owing to the high workload. The analyses showed no significant differences in any of the baseline outcomes or demographic variables between those who dropped out early and those who continued throughout the program. Module 1 was completed by 82% (50/61) in the W-iCBT and 93.4% (57/61) iCBT groups, module 2 by 75.4% (46/61) and 90.2% (55/61), module 3 by 78.7% (48/61) and 91.8% (56/61), module 4 by 72.1% (44/61) and 90.2% (55/61), module 5 by 70.5% (43/61) and 88.5% (54/61), module 6 by 65.6% (40/61) and 86.9% (53/61), module 7 by 65.6% (40/61) and 83.6% (51/61), module 8 by 63.9% (39/61) and 77.0% (47/61), module 9 by 62.3% (38/61) and 67.2% (41/61), and module 10 by 60.7% (37/61) and 67.2% (41/61) of the participants, respectively.

### Client Satisfaction

Client utility and satisfaction were assessed on a 5-point scale (1=low satisfaction to 5=high satisfaction). The utility was given an average score of 4.18 (SD 0.72) in the W-iCBT group and 4.16 (SD 0.86) in the iCBT group, and satisfaction was given a score of 4.50 (SD 0.97) and 4.24 (SD 0.76) for each group. Only 2 participants (in the iCBT group) were hesitant about whether they would recommend the program.

### Primary Outcome Analyses

The means and SDs for all groups for the primary outcomes are presented in Table 3. As depicted in Table 4, the repeated measures ANOVA for the primary outcome, the SMBQ, revealed a significant overall effect (*F*_4,358_=5.39; *P<*.001) between the interventions (W-iCBT and generic iCBT) and WLC. In the following separate ANOVA, both the W-iCBT and iCBT showed lower scores on the primary outcome SMBQ at posttest (T2; *F*_2,179_=14.9; *P<*.001) and at the 6 months follow-up (T3; *F*_2,179_=7.47; *P<*.01) than the WLC. Large effect sizes of Cohen *d* were observed at the posttest (W-iCBT: Cohen *d*=1.00; 95% CI 0.57-1.43 and iCBT: Cohen *d*=0.83; 95% CI 0.41-1.25) and at the 6 months follow-up (W-iCBT: Cohen *d*=0.74; 95% CI 0.30-1.18 and iCBT: Cohen *d*=0.74; 95% CI 0.35-1.13). The repeated measures ANOVA found no significant differences between the 2 interventions at any time point on the primary outcome (SMBQ T1-T3; *F*_1,120_=0.019; *P=*.99).

**Table 4:** Results of the repeated measures ANOVA and Cohen d for the primary and secondary outcome measures (intention-to-treat [ITT] sample) at posttest (T2) and 6 months follow-up (T3) time points.

Outcome			ANOVA overall effect				T2 between-groups effect										T3 between-groups effect												
			*F* test (*df*=4,358)		*P* value		W-iCBT^a^					iCBT^b^					W-iCBT					iCBT							
							Cohen *d*		95% CI		Cohen *d*			95% CI		Cohen *d*			95% CI		Cohen *d*			95% CI					
**Primary outcome**																													
	**Burnout (1-7)^c^**			5.39		<.001		1.00^d^		0.57 to 1.43			0.83^d^		0.41 to 1.25			0.74^d^		0.30 to 1.18			0.74^d^		0.35 to 1.13				
		Emotional fatigue		4.99		<.001		0.78^d^		0.39 to 1.17			0.71^d^		0.27 to 1.22			0.79^d^		0.41 to 1.17			0.74^d^		0.26 to 1.22				
		Cognitive weariness		4.92		<.001		0.91^d^		0.51 to 1.32			0.90^d^		0.47 to 1.33			0.70^d^		0.30 to 1.11			0.84^d^		0.45 to 1.23				
		Tension		4.58		<.001		0.84^d^		0.40 to 1.27			0.73^d^		0.33 to 1.12			0.54^e^		0.12 to 0.95			0.44^e^		0.06 to 0.82				
		Listlessness		5.16		<.001		0.88^d^		0.48 to 1.29			0.73^d^		0.34 to 1.11			0.47^e^		0.04 to 0.91			0.49^e^		0.11 to 0.87				
**Health related**																													
	Perceived stress (0-40)^f^			4.85		<.001		0.85^d^		0.44 to 1.26			0.79^d^		0.36 to 1.22			0.77^g^		0.35 to 1.19			0.68^g^		0.16 to 1.21				
	Exhaustion (0-54)^h^			4.46		<.001		0.87^d^		0.41 to 1.32			0.87^d^		0.42 to 1.31			0.50^e^		0.08 to 0.93			0.60^g^		0.14 to 1.06				
	Depression (0-54)^i^			4.38		.002		0.84^d^		0.43 to 1.24			0.81^d^		0.43 to 1.20			0.49^e^		0.07 to 0.92			0.51^e^		0.06 to 0.96				
	Anxiety (0-21)^j^			3.60		.008		0.70^d^		0.28 to 1.12			0.72^d^		0.34 to 1.10			0.42^e^		0.05 to 0.79			0.17		0.21 to 0.55				
	Insomnia (0-28)^k^			7.44		<.001		0.87^d^		0.44 to 1.30			0.92^d^		0.49 to 1.34			0.55^e^		0.13 to 0.97			0.60^g^		0.19 to 1.01				
	Alcohol (0-40)^l^			0.06		.99		0.13		0.25 to 0.51			0.03		0.42 to 0.35			N/A^m^		N/A			N/A		N/A				
	Quality of life (0-30)^n^			2.42		.06		0.50^g^		0.11 to 0.89			0.43^e^		0.06 to 0.91			0.59^g^		0.19 to 0.99			0.67^g^		0.27 to 1.07				
**Work related**																													
	Work experience (32-192)^o^			1.40		.26		0.16		−0.24 to 0.56			0.20		−0.19 to 0.59			0.28		−0.17 to 0.74			0.37		−0.07 to 0.81				
	Work ability (7-49)^p^			1.18		.32		0.40^e^		0.03 to 0.77			0.36		−0.01 to 0.73			0.22		−0.22 to 0.66			0.20		−0.21 to 0.60				
	**Recovery (4-80)^q^**			2.94		.03		0.89^d^		1.28 to 0.50			0.88^d^		0.47 to 1.30			0.64^g^		0.23 to 1.05			0.53^g^		0.15 to 0.91				
		Psychological detach		2.55		.04		0.93^d^		0.51 to 1.34			0.71^d^		0.32 to 1.11			0.50^e^		0.01 to 1.00			0.43^e^		0.05 to 0.82				
		Relaxation		5.07		<.001		0.96^d^		0.54 to 1.39			0.81^d^		0.42 to 1.20			0.59^g^		0.18 to 1.01			0.53^e^		0.10 to 0.97				
		Mastery		2.30		.06		0.58^g^		0.20 to 0.96			0.52^g^		0.13 to 0.91			0.62^g^		0.18 to 1.06			0.49^e^		0.07 to 0.90				
		Control		1.53		.20		0.53^g^		0.11 to 0.94			0.68^d^		0.30 to 1.06			0.37		−0.02 to 0.76			0.48^e^		0.08 to 0.88				

^a^W-iCBT: work-focused and internet-based cognitive behavioral therapy.

^b^iCBT: internet-based cognitive behavioral therapy.

^c^SMBQ: Shirom-Melamed Burnout Questionnaire.

^d^*P*<.001.

^e^*P*<.05.

^f^PSS-10: Perceived Stress Scale.

^g^*P*<.01.

^h^KEDS: Karolinska Exhaustion Disorder Scale.

^i^MADRS-S: Montgomery-Åsberg Depression Rating Scale–Self-rating version.

^j^GAD-7: Generalized Anxiety Disorder 7-item scale.

^k^ISI: Insomnia Severity Index.

^l^AUDIT: Alcohol Use Disorders Identification Test.

^m^N/A: not applicable.

^n^SDS: Sheehan Disability Scale.

^o^WEMS: Work Experience Measurement Scale.

^p^WAI: Work Ability Index.

^q^REQ: Recovery Experience Questionnaire.

### Secondary Outcome Analysis

The means and SDs for all groups of secondary outcomes are presented in Table 3. Table 4 presents the results of the ITT analyses of the secondary outcomes. The repeated measures ANOVA found significant overall effects in favor of the 2 intervention groups for all outcomes at T2 and T3, apart from work experience (*F*_4,358_=1.40; *P=*.24) and work ability (*F*_4,358_=1.18; *P=*.32). In the following analyses of simple effects, we found significant improvement in the W-iCBT group, when compared with the WLC group, on work ability (T2: *F*_2,179_=4.61; *P=*.03 and T3: *F*_2,179_=1.87; *P=*.18) and SA (T2: H_2_=−23.58; *P=*.01 and T3: H_2_=−18.44; *P=*.03). At the 6-month follow-up, SA was 324 days lower in the W-iCBT group (median 0.00; *R*=66; H_2_=−18.43; *P=*.03) than in the iCBT group and 445 days (median 2.00; *R*=70; H_2_=−18.44; *P=*.03) lower than in the WLC group (median 3.00; *R*=77). However, no significant differences were found in the net days of long-term sick leave between any groups (H_2_=−0.82; *P=*.66). The total net days on benefits owing to long-term sick leave were 1932 days in the W-iCBT group, 2328 days in the iCBT group, and 2435 days in the WLC group. Accordingly, 14 participants in the W-iCBT group were on long-term sick leave at T2 (6 ended and 3 started) and 9 participants at T3 (5 ended and 0 started). Corresponding values for the iCBT and WLC groups at T2 were 17 and 15 participants, respectively (iCBT: 4 ended and 4 started; WLC: 5 ended and 3 started), and 11 and 10 participants, respectively, at T3 (iCBT: 8 ended and 2 started; WLC: 10 ended and 5 started).

### Long-term Follow-up

The mean scores in the primary and secondary outcomes were maintained or continued to improve in both intervention groups at the 12-month follow-up. Significant differences between the iCBT and W-iCBT groups (Multimedia Appendix 1) were only seen on the SMBQ subscale tension (*F*_1,688_=5.80; *P=*.02) and REQ subscale psychological detachment (*F*_1,688_=6.11; *P=*.01).

### Intervention Support

Participants received an equal amount of time (minutes per week) for support (W-iCBT: mean 12.11, SD 7.76; iCBT: mean 12.92, SD 7.08; *F*_1,120_=0.356; *P=*.55). In addition, the participants were asked questions about how they perceived the support. Overall, 90% (55/61) in the W-iCBT group and 96% (59/182) in the iCBT group experienced the support as relevant and helpful.

### Completers-Only Analyses

Completers-only analysis revealed significant (*P*<.001) and larger effects for the primary outcome (SMBQ) at postassessment time point (W-iCBT: Cohen *d*=1.31; 95% CI 0.86-1.77 and iCBT: Cohen *d*=1.13; 95% CI 0.71-1.55) and at the 6-month follow-up (W-iCBT: Cohen *d*=0.98; 95% CI 0.53-1.43 and iCBT: Cohen *d*=0.88; 95% CI 0.46-1.30) compared with the ITT-analyses. Significant differences and larger effect sizes were also observed in the secondary outcomes (data not shown).

### Clinically Significant Change

The number of participants fulfilling the criteria for clinically significant change on the ITT data on the SMBQ at postassessment time point was 56% (34/61) in the W-iCBT group, 47% (29/61) in the iCBT group, and 21% (13/60) in the WLC group. At the 6 months follow-up, the proportion of clinically significant changes were W-CBT, 47% (29/61), iCBT, 48% (30/61), and WLC, 37% (22/60), respectively. On the KEDS, the proportions were 26% (16/61) in the W-iCBT group, 23% (14/61) in the iCBT group, and 7% (4/60) in the WLC group; at the 6 months follow-up, they were W-CBT, 34% (21/61); iCBT, 28% (17/61); and WLC, 11% (7/60).

## Discussion

### Principal Findings

To the best of our knowledge, this study is the first trial examining a work-focused intervention and a generic internet-based intervention in a clinical sample of employees with stress-related disorders. The results confirmed the primary hypothesis that both interventions were equally effective in reducing symptoms of perceived stress, burnout, exhaustion, depression, anxiety, and insomnia and in improving recovery from work and quality of life compared with a WLC group. Secondary explorative analyses indicated positive effects on work ability and a reduction in the number of days of SA in the work-focused group. No significant effects were found on outcomes for alcohol use, work experience, or net days on the benefits for long-term sick leave.

The effects found on health-related outcomes were larger than those previously reported in a meta-analysis of internet-based stress management trials [27]: perceived stress, Cohen *d*=0.43; depression, Cohen *d*=0.34; and anxiety, Cohen *d*=0.32. One plausible explanation may be that previous internet-based studies of interventions to reduce stress have largely included individuals with lower symptom severity (nonclinical). There are indications that greater initial symptom severity results in higher response and remission rates [77,78].

Most of the participants (W-iCBT: 42/61, 69%; iCBT: 41/61, 67%) in the intervention groups fulfilled the criteria for clinically significant change in the primary outcome, SMBQ, and were maintained at the 6-month follow-up. The proportion of clinically significant changes was comparable with previous trials [33,76]. However, there was a considerable discrepancy in the number of participants who achieved clinically significant changes when measured using KEDS compared with SMBQ. This might reflect that SMBQ and KEDS measure different underlying constructs, as noted in previous research [79].

As hypothesized, changes in health- and work-related outcomes remained stable in both intervention groups at the 12-month follow-up. The results were in line with the long-term effects found in a meta-analysis of internet-based stress management trials [27], which showed moderate effect sizes (Cohen *d*=0.56) up to 6 months after the treatment. These results are encouraging as they provide further evidence of the long-term benefits of relativity short iCBT interventions. However, we still struggle with the fact that about one-third of patients relapse or continue to experience residual symptoms several years after treatment for stress-related disorders [80]. Future studies should examine the use of minimally invasive long-term remote patient monitoring to further extend the long-term effects of iCBT stress interventions.

Interestingly, the effects on work ability and SA were only seen between the W-iCBT and WLC groups. However, these effects were small but comparable with those found in a meta-analysis of psychological interventions for individuals in SA because of common mental disorders [19]: Hedges *g*=0.22 for work-focused CBT interventions. These results are promising because SA has direct effects on people’s well-being and leads to large costs for society [16]. Although effects on SA were present in the W-iCBT group, no significant effects were present in any group with regard to net days on the benefits of long-term sick leave. One possible explanation could be that the 2 outcomes were assessed differently. SA was conceptualized as the self-rated number of days absent from work during the past 3 months while being physically or mentally ill and measured at 3 time points: pretreatment, posttreatment, and 6-month follow-up. However, long-term sick leave was based on data from the Swedish Social Insurance Agency on the number of net days on sickness benefit between the pretreatment and 6-month follow-up assessments. In Sweden, sickness benefits from the Swedish Social Insurance Agency are due from day 15 on sick leave. Thus, absence during the first 14 days of illness was not included in the analysis of this outcome. Accordingly, SA and long-term sick leave were assessed differently, with different starting points, conceptualizations, and time intervals.

Inspired by the recovery from work training by Hahn et al [41,71], we included modules corresponding to the subdimensions of the REQ, namely, psychological detachment, relaxation, mastery, and control. The effect in this study was larger compared with previous internet-based stress management studies, including recovery techniques and the REQ [46,81,82]. Generally, recovery is a component of psychological treatment for stress and burnout. However, few studies have focused exclusively on recovery training. Consequently, it would be interesting to develop and evaluate an internet-based recovery training program, which exclusively focuses on various recovery skills. Hopefully, this can be an accessible and successful way to prevent stress-related problems in the working population.

This study has several limitations. First, although this study focused on recruiting participants who were on sick leave because of stress and burnout, only 51 (28%) of 182 received sickness benefits at T1 and T3, resulting in unsatisfactory power in the statistical analysis. It is possible that the use of an open recruitment strategy and the fact that the intervention was delivered from an external institution (the university) may have had an impact on the recruitment. Future studies could use another recruitment and delivery approach, for example, directly via primary and occupational health care, to include more participants who are on sick leave because of stress-related illness. Second, by using an open recruitment strategy, we cannot rule out the risk of potential selection bias, selecting those cases that are most motivated to participate and willing to change. For example, 92% (167/182) of the participants had a university-level educational background, compared with 28% in the general population [83], and 62% (113/182) were working in the social, health care, or education sector. Therefore, future studies that include participants that are more representative of the general working population are needed. Providing W-iCBT directly, integrated into the workplace, could lower thresholds and be a successful approach in including various employees from different industries. Third, the overall study attrition rate was moderate; however, it was twice as large in W-iCBT (T2: 18/61, 30%; T3: 22/61, 36%) compared with generic iCBT (T2: 9/61, 15%; T3: 11/61, 18%). Consistent with previous research [36], this might be due to the work-focused content (3 regular pages including text and corresponding worksheets and homework assignments) included in the W-iCBT and the extra workload relative to the generic iCBT intervention, constituting a potential stressor. Perhaps the individualization and integration of the work-focused content could further streamline the treatment protocol, increase adherence, and prevent dropout. Fourth, we did not include any mediator or moderator analysis. Hence, future studies should be designed with repeated assessments to test for mediating and moderating mechanisms. For instance, it would be interesting to examine the mediation role of recovery in interventions for stress-related disorders. Fifth, none of the interventions examined in this trial included workplace involvement. Workplace dialogue and involvement (eg, manager, human resource professionals) were only encouraged indirectly through the participants. Workplace involvement is an important factor for returning to work [13,84]. Hence, it would be interesting to evaluate an internet-based and work-focused program for employees experiencing stress-related disorders with a parallel program including workplace involvement (eg, managerial support and perspective on stress, burnout, recovery, and RTW).

### Conclusions

This trial provides further evidence of the efficacy of internet-based interventions in a clinical sample of employees experiencing stress-related disorders. To our knowledge, this was the first internet-based trial integrating a work-focused format, with effects on important work-related outcomes such as SA and work ability. These preliminary results are promising, indicating that treatments that include work aspects may have the potential to accelerate recovery and reduce short-term SA because of stress-related disorders. Nonetheless, further research is needed to investigate the potential of internet-based and work-focused interventions.

## Appendix

Multimedia Appendix 1 Results of the ANOVA and Cohen d for the primary and secondary outcome measures (intention to treat sample) between the work-focused and internet-based cognitive behavioral therapy and internet-based cognitive behavioral therapy at the 12 months follow-up (T4).

Multimedia Appendix 2 CONSORT-eHEALTH checklist (V 1.6.1).

## Abbreviations

AUDIT: Alcohol Use Disorders Identification Test
CONSORT: Consolidated Standards of Reporting Trials
DSM-5: Diagnostic and Statistical Manual of Mental Disorders, Fifth Edition
ED: exhaustion disorder
GAD-7: Generalized Anxiety Disorder 7-item Scale
iCBT: internet-based cognitive behavioral therapy
ICD-10: International Statistical Classification of Diseases and Related Health Problems, 10th Revision
ISI: Insomnia Severity Index
ITT: intention to treat
KEDS: Karolinska Exhaustion Disorder Scale
MADRS-S: Montgomery-Åsberg Depression Rating Scale–Self-rating version
OECD: Organisation for Economic Co-operation and Development
PSS-10: Perceived Stress Scale
REQ: Recovery Experience Questionnaire
RTW: return to work
SA: sickness absence
SDS: Sheehan Disability Scale
SMBQ: Shirom-Melamed Burnout Questionnaire
TiC-P: Trimbos and Institute of Medical Technology Assessment Cost Questionnaire for Psychiatry
WAI: Work Ability Index
WEMS: Work Experience Measurement Scale.
W-iCBT: work-focused and internet-based cognitive behavioral therapy
WLC: waitlist control
