# Supplementary material for: Work-Focused Versus Generic Internet-Based Interventions for Employees With Stress-Related Disorders: Randomized Controlled Trial
Source: J Med Internet Res. 2023 Apr 25;25:e34446. doi: 10.2196/34446 (PMC10170369; doi:10.2196/34446)
Supplement: Multimedia Appendix 1 [file jmir_v25i1e34446_app1.docx]

Multimedia Appendix 1

Table 5. Results of the analysis of variances (ANOVA) and Cohen’s d for the primary and secondary outcome measures (ITT sample) between the W-iCBT and iCBT at the 12 months follow-up (T4). [95% CI=95% confidence interval.]

| Outcome | ANOVA ^a^ | |
| --- | --- | --- |
|  | F (1,688) | P |
|  |  |  |
| **Primary outcome** |  |  |
| Burnout (1-7) ^b^ | 0.076 | 0.783 |
| - Emotional fatigue | 0.976 | 0.324 |
| - Cognitive weariness | 0.998 | 0.318 |
| - Tension | 5.804 | 0.016 |
| - Listlessness | 0.172 | 0.678 |
| **Health related** |  |  |
| Perceived stress (0-40) ^c^ | 1.695 | 0.193 |
| Exhaustion (0-54) ^d^ | 0.242 | 0.623 |
| Depression (0-54) ^e^ | 1.483 | 0.224 |
| Anxiety (0-21) ^f^ | 3.358 | 0.067 |
| Insomnia (0-28) ^g^ | 2.254 | 0.134 |
| Quality of life (0-30) ^h^ | 0.531 | 0.466 |
| **Work related** |  |  |
| Work experience (32-192) ^i^ | 3.621 | 0.057 |
| Work ability (7-49) ^j^ | 3.320 | 0.069 |
| Recovery (4-80) ^k^ | 0.110 | 0.740 |
| - Psychological detachment | 6.107 | 0.014 |
| - Relaxation | 1.279 | 0.258 |
| - Mastery | 0.506 | 0.477 |
| - Control | 1510 | 0.220 |

^a^ Missing data imputed by multiple imputation

^b^ SMBQ=Shirom-Melamed Burnout Questionnaire

^c^ PSS-10=Perceived Stress Scale

^d^ KEDS=Karolinska Exhaustion Disorder Scale

^e^ MADRS-S=Montgomery Åsberg Depression Rating Scale-self-assessment

^f^ GAD-7=Generalized Anxiety Disorder 7-item scale

^g^ ISI=Insomnia Severity Index

^h^ SDS=Sheehan Disability Scale

^i^ WEMS=Work Experience Measurement Scale

^j^ WAI=Work Ability Index

^k^ REQ=Recovery Experience Questionnaire
