# Supplementary material for: Work-Focused Versus Generic Internet-Based Interventions for Employees With Stress-Related Disorders: Randomized Controlled Trial
Source: J Med Internet Res. 2023 Apr 25;25:e34446. doi: 10.2196/34446 (PMC10170369; doi:10.2196/34446)
Supplement: Multimedia Appendix 2 [file jmir_v25i1e34446_app2.pdf]

CONSORT-EHEALTH (V 1.6.1) - Submission/Publication Form

The CONSORT-EHEALTH checklist is intended for authors of randomized trials evaluating web-based and Internet-based applications/interventions, including mobile interventions, electronic games (incl multiplayer games), social media, certain telehealth applications, and other interactive and/or networked electronic applications. Some of the items (e.g. all subitems under item 5 - description of the intervention) may also be applicable for other study designs.

The goal of the CONSORT EHEALTH checklist and guideline is to be
a) a guide for reporting for authors of RCTs,
b) to form a basis for appraisal of an ehealth trial (in terms of validity)

CONSORT-EHEALTH items/subitems are MANDATORY reporting items for studies published in the Journal of Medical Internet Research and other journals / scientific societies endorsing the checklist.

Items numbered 1., 2., 3., 4a., 4b etc are original CONSORT or CONSORT-NPT (non-pharmacologic treatment) items.
Items with Roman numerals (i., ii, iii, iv etc.) are CONSORT-EHEALTH extensions/clarifications.

As the CONSORT-EHEALTH checklist is still considered in a formative stage, we would ask that you also RATE ON A SCALE OF 1-5 how important/useful you feel each item is FOR THE PURPOSE OF THE CHECKLIST and reporting guideline (optional).

Mandatory reporting items are marked with a red *.
In the textboxes, either copy & paste the relevant sections from your manuscript into this form - please include any quotes from your manuscript in QUOTATION MARKS,
or answer directly by providing additional information not in the manuscript, or elaborating on why the item was not relevant for this study.

YOUR ANSWERS WILL BE PUBLISHED AS A SUPPLEMENTARY FILE TO YOUR PUBLICATION IN JMIR AND ARE CONSIDERED PART OF YOUR PUBLICATION (IF ACCEPTED).
Please fill in these questions diligently. Information will not be copyedited, so please use proper spelling and grammar, use correct capitalization, and avoid abbreviations.

DO NOT FORGET TO SAVE AS PDF _AND_ CLICK THE SUBMIT BUTTON SO YOUR ANSWERS ARE IN OUR DATABASE !!!

Citation Suggestion (if you append the pdf as Appendix we suggest to cite this paper in the caption):
Eysenbach G, CONSORT-EHEALTH Group
CONSORT-EHEALTH: Improving and Standardizing Evaluation Reports of Web-based and Mobile Health Interventions
J Med Internet Res 2011;13(4):e126

URL: http://www.jmir.org/2011/4/e126/
doi: 10.2196/jmir.1923
PMID: 22209829

robert@healo.app (not shared) Switch accounts

Draft not saved

*Required

Your name *

First Last

Robert Persson Asplund

Primary Affiliation (short), City, Country *

University of Toronto, Toronto, Canada

Linköping University

Your e-mail address *

abc@gmail.com

robert.persson.asplund@liu.se

Title of your manuscript *

Provide the (draft) title of your manuscript.

Work-Focused Versus Generic Internet-Based Interventions for Employees With Stress-Related Disorders: Randomized Controlled Trial

Name of your App/Software/Intervention *

If there is a short and a long/alternate name, write the short name first and add the long name in brackets.

iStress & iTherapy

Evaluated Version (if any)

e.g. "V1", "Release 2017-03-01", "Version 2.0.27913"

Your answer

Language(s) *

What language is the intervention/app in? If multiple languages are available, separate by comma (e.g. "English, French")

Swedish

URL of your Intervention Website or App

e.g. a direct link to the mobile app on app in appstore (itunes, Google Play), or URL of the website. If the intervention is a DVD or hardware, you can also link to an Amazon page.

www.istress.se

URL of an image/screenshot (optional)

Your answer

Accessibility *

Can an enduser access the intervention presently?

- ( ) access is free and open
- ( ) access only for special usergroups, not open
- ( ) access is open to everyone, but requires payment/subscription/in-app purchases
- (•) app/intervention no longer accessible
- ( ) Other:

Primary Medical Indication/Disease/Condition *

e.g. "Stress", "Diabetes", or define the target group in brackets after the condition, e.g. "Autism (Parents of children with)", "Alzheimers (Informal Caregivers of)"

Adjustment disorders

Primary Outcomes measured in trial *

comma-separated list of primary outcomes reported in the trial

Shirom-Melamed Burnout Questionnaire

Secondary/other outcomes

Are there any other outcomes the intervention is expected to affect?

Perceived Stress Scale, Karolinska Exhaustion Disorder Scale, Montgomery-Åsberg Depression Rating Scale–Self-rating version, Generalized Anxiety Disorder 7-item scale, Insomnia Severity Index, Alcohol Use Disorders Identification Test, Sheehan Disability Scale, Work Experience Measurement Scale, Work Ability Index, Recovery Experience Questionnaire

Recommended "Dose" *

What do the instructions for users say on how often the app should be used?

- (•) Approximately Daily
- ( ) Approximately Weekly
- ( ) Approximately Monthly
- ( ) Approximately Yearly
- ( ) "as needed"
- ( ) Other:

Approx. Percentage of Users (starters) still using the app as recommended after 3 months *

- ( ) unknown / not evaluated
- ( ) 0-10%
- ( ) 11-20%
- (•) 21-30%
- ( ) 31-40%
- ( ) 41-50%
- ( ) 51-60%
- ( ) 61-70%
- ( ) 71%-80%
- ( ) 81-90%
- ( ) 91-100%
- ( ) Other:

Overall, was the app/intervention effective? *

- (selected) yes: all primary outcomes were significantly better in intervention group vs control
- partly: SOME primary outcomes were significantly better in intervention group vs control
- no statistically significant difference between control and intervention
- potentially harmful: control was significantly better than intervention in one or more outcomes
- inconclusive: more research is needed
- Other:

Article Preparation Status/Stage *

At which stage in your article preparation are you currently (at the time you fill in this form)

- not submitted yet - in early draft status
- not submitted yet - in late draft status, just before submission
- submitted to a journal but not reviewed yet
- submitted to a journal and after receiving initial reviewer comments
- (selected) submitted to a journal and accepted, but not published yet
- published
- Other:

Journal *

If you already know where you will submit this paper (or if it is already submitted), please provide the journal name (if it is not JMIR, provide the journal name under "other")

- ○ not submitted yet / unclear where I will submit this
- ● Journal of Medical Internet Research (JMIR)
- ○ JMIR mHealth and UHealth
- ○ JMIR Serious Games
- ○ JMIR Mental Health
- ○ JMIR Public Health
- ○ JMIR Formative Research
- ○ Other JMIR sister journal
- ○ Other:

Is this a full powered effectiveness trial or a pilot/feasibility trial? *

- ○ Pilot/feasibility
- ● Fully powered

Manuscript tracking number *

If this is a JMIR submission, please provide the manuscript tracking number under "other" (The ms tracking number can be found in the submission acknowledgement email, or when you login as author in JMIR. If the paper is already published in JMIR, then the ms tracking number is the four-digit number at the end of the DOI, to be found at the bottom of each published article in JMIR)

- ○ no ms number (yet) / not (yet) submitted to / published in JMIR
- ● Other: (J Med Internet Res 2023;25:e34446) doi: 10.2196/34446

TITLE AND ABSTRACT

1a) TITLE: Identification as a randomized trial in the title

1a) Does your paper address CONSORT item 1a? *

I.e does the title contain the phrase "Randomized Controlled Trial"? (if not, explain the reason under "other")

- (●) yes
- ( ) Other:

1a-i) Identify the mode of delivery in the title

Identify the mode of delivery. Preferably use “web-based” and/or “mobile” and/or “electronic game” in the title. Avoid ambiguous terms like “online”, “virtual”, “interactive”. Use “Internet-based” only if Intervention includes non-web-based Internet components (e.g. email), use “computer-based” or “electronic” only if offline products are used. Use “virtual” only in the context of “virtual reality” (3-D worlds). Use “online” only in the context of “online support groups”. Complement or substitute product names with broader terms for the class of products (such as “mobile” or “smart phone” instead of “iphone”), especially if the application runs on different platforms.

subitem not at all important

- 1 ( )
- 2 ( )
- 3 (●)
- 4 ( )
- 5 ( )

essential

Clear selection

Does your paper address subitem 1a-i? *

Copy and paste relevant sections from manuscript title (include quotes in quotation marks "like this" to indicate direct quotes from your manuscript), or elaborate on this item by providing additional information not in the ms, or briefly explain why the item is not applicable/relevant for your study

Yes, we have adressed this criteria.

1a-ii) Non-web-based components or important co-interventions in title

Mention non-web-based components or important co-interventions in title, if any (e.g., “with telephone support”).

subitem not at all important

- 1 (selected)
- 2
- 3
- 4
- 5

essential

Clear selection

Does your paper address subitem 1a-ii?

Copy and paste relevant sections from manuscript title (include quotes in quotation marks "like this" to indicate direct quotes from your manuscript), or elaborate on this item by providing additional information not in the ms, or briefly explain why the item is not applicable/relevant for your study

Not applicable/relevant for our study

1a-iii) Primary condition or target group in the title

Mention primary condition or target group in the title, if any (e.g., “for children with Type I Diabetes”) Example: A Web-based and Mobile Intervention with Telephone Support for Children with Type I Diabetes: Randomized Controlled Trial

subitem not at all important

1 ○

2 ○

3 ○

4 ○

5 ◉

essential

Clear selection

Does your paper address subitem 1a-iii? *

Copy and paste relevant sections from manuscript title (include quotes in quotation marks "like this" to indicate direct quotes from your manuscript), or elaborate on this item by providing additional information not in the ms, or briefly explain why the item is not applicable/relevant for your study

Term stress-related disorders is included in the title and manuscript.

1b) ABSTRACT: Structured summary of trial design, methods, results, and conclusions

NPT extension: Description of experimental treatment, comparator, care providers, centers, and blinding status.

1b-i) Key features/functionalities/components of the intervention and comparator in the METHODS section of the ABSTRACT

Mention key features/functionalities/components of the intervention and comparator in the abstract. If possible, also mention theories and principles used for designing the site. Keep in mind the needs of systematic reviewers and indexers by including important synonyms. (Note: Only report in the abstract what the main paper is reporting. If this information is missing from the main body of text, consider adding it)

subitem not at all important

- 1 (selected)
- 2
- 3
- 4
- 5

essential

Clear selection

Does your paper address subitem 1b-i? *

Copy and paste relevant sections from the manuscript abstract (include quotes in quotation marks "like this" to indicate direct quotes from your manuscript), or elaborate on this item by providing additional information not in the ms, or briefly explain why the item is not applicable/relevant for your study

Not applicable/relevant for our study

1b-ii) Level of human involvement in the METHODS section of the ABSTRACT

Clarify the level of human involvement in the abstract, e.g., use phrases like "fully automated" vs. "therapist/nurse/care provider/physician-assisted" (mention number and expertise of providers involved, if any). (Note: Only report in the abstract what the main paper is reporting. If this information is missing from the main body of text, consider adding it)

subitem not at all important

1 ○

2 ○

3 ◉

4 ○

5 ○

essential

Clear selection

Does your paper address subitem 1b-ii?

Copy and paste relevant sections from the manuscript abstract (include quotes in quotation marks "like this" to indicate direct quotes from your manuscript), or elaborate on this item by providing additional information not in the ms, or briefly explain why the item is not applicable/relevant for your study

We have provided information in the manuscript (not abstract) regering provider support/guidence.

1b-iii) Open vs. closed, web-based (self-assessment) vs. face-to-face assessments in the METHODS section of the ABSTRACT

Mention how participants were recruited (online vs. offline), e.g., from an open access website or from a clinic or a closed online user group (closed usergroup trial), and clarify if this was a purely web-based trial, or there were face-to-face components (as part of the intervention or for assessment). Clearly say if outcomes were self-assessed through questionnaires (as common in web-based trials). Note: In traditional offline trials, an open trial (open-label trial) is a type of clinical trial in which both the researchers and participants know which treatment is being administered. To avoid confusion, use “blinded” or “unblinded” to indicated the level of blinding instead of “open”, as “open” in web-based trials usually refers to “open access” (i.e. participants can self-enrol). (Note: Only report in the abstract what the main paper is reporting. If this information is missing from the main body of text, consider adding it)

subitem not at all important

1 ○

2 ○

3 ○

4 ○

5 ◉

essential

Clear selection

Does your paper address subitem 1b-iii?

Copy and paste relevant sections from the manuscript abstract (include quotes in quotation marks "like this" to indicate direct quotes from your manuscript), or elaborate on this item by providing additional information not in the ms, or briefly explain why the item is not applicable/relevant for your study

Methods: In this trial, 182 employees, mainly employed in the health care, IT, or educational sector, who fulfilled the criteria for a stress-related disorder, were randomized to a 10-week W-iCBT (n=61, 33.5%), generic iCBT (n=61, 33.5%), or WLC (n=60, 33%). Self-rated questionnaires on perceived stress, burnout, exhaustion, and other mental health– and work-related outcomes were administered before and after the treatment and at 6- and 12-month follow-ups.

1b-iv) RESULTS section in abstract must contain use data

Report number of participants enrolled/assessed in each group, the use/uptake of the intervention (e.g., attrition/adherence metrics, use over time, number of logins etc.), in addition to primary/secondary outcomes. (Note: Only report in the abstract what the main paper is reporting. If this information is missing from the main body of text, consider adding it)

subitem not at all important

1 ○

2 ○

3 ○

4 ○

5 ◉

essential

Clear selection

Does your paper address subitem 1b-iv?

Copy and paste relevant sections from the manuscript abstract (include quotes in quotation marks "like this" to indicate direct quotes from your manuscript), or elaborate on this item by providing additional information not in the ms, or briefly explain why the item is not applicable/relevant for your study

Methods: In this trial, 182 employees, mainly employed in the health care, IT, or educational sector, who fulfilled the criteria for a stress-related disorder, were randomized to a 10-week W-iCBT (n=61, 33.5%), generic iCBT (n=61, 33.5%), or WLC (n=60, 33%). Self-rated questionnaires on perceived stress, burnout, exhaustion, and other mental health– and work-related outcomes were administered before and after the treatment and at 6- and 12-month follow-ups.

1b-v) CONCLUSIONS/DISCUSSION in abstract for negative trials

Conclusions/Discussions in abstract for negative trials: Discuss the primary outcome - if the trial is negative (primary outcome not changed), and the intervention was not used, discuss whether negative results are attributable to lack of uptake and discuss reasons. (Note: Only report in the abstract what the main paper is reporting. If this information is missing from the main body of text, consider adding it)

subitem not at all important

- 1 (selected)
- 2
- 3
- 4
- 5

essential

Clear selection

Does your paper address subitem 1b-v?

Copy and paste relevant sections from the manuscript abstract (include quotes in quotation marks "like this" to indicate direct quotes from your manuscript), or elaborate on this item by providing additional information not in the ms, or briefly explain why the item is not applicable/relevant for your study

Not applicable/relevant for our study

INTRODUCTION

2a) In INTRODUCTION: Scientific background and explanation of rationale

2a-i) Problem and the type of system/solution

Describe the problem and the type of system/solution that is object of the study: intended as stand-alone intervention vs. incorporated in broader health care program? Intended for a particular patient population? Goals of the intervention, e.g., being more cost-effective to other interventions, replace or complement other solutions? (Note: Details about the intervention are provided in “Methods” under 5)

subitem not at all important

1 ○

2 ○

3 ◉

4 ○

5 ○

essential

Clear selection

Does your paper address subitem 2a-i? *

Copy and paste relevant sections from the manuscript (include quotes in quotation marks "like this" to indicate direct quotes from your manuscript), or elaborate on this item by providing additional information not in the ms, or briefly explain why the item is not applicable/relevant for your study

Purpose of This Study
The aim of this study was to evaluate the efficacy of a work-focused iCBT (W-iCBT) intervention compared with generic iCBT and a waitlist control (WLC) group in a self-referred sample of employees with stress-related disorders.

2a-ii) Scientific background, rationale: What is known about the (type of) system

Scientific background, rationale: What is known about the (type of) system that is the object of the study (be sure to discuss the use of similar systems for other conditions/diagnoses, if appropiate), motivation for the study, i.e. what are the reasons for and what is the context for this specific study, from which stakeholder viewpoint is the study performed, potential impact of findings [2]. Briefly justify the choice of the comparator.

subitem not at all important

- 1 ○
- 2 ○
- 3 ○
- 4 ◉
- 5 ○

essential

Clear selection

Does your paper address subitem 2a-ii? *

Copy and paste relevant sections from the manuscript (include quotes in quotation marks "like this" to indicate direct quotes from your manuscript), or elaborate on this item by providing additional information not in the ms, or briefly explain why the item is not applicable/relevant for your study

Despite the well-documented efficacy of stress management interventions [17], and some promising results of work-focused interventions [20-25], the range of interventions is not proportionate to the needs of distressed employees [26]. This clarifies the need to further develop and evaluate work-directed interventions that are accessible to the working population.

Studies suggest that stress can be managed through internet- and computer-based interventions [27-30]. In a meta-analysis [27] including 26 controlled studies (n=4226), small to moderate effects were found on the outcomes of stress (Cohen d=0.43), depression (Cohen d=0.34), and anxiety (Cohen d=0.32). Subgroup analyses revealed that guided interventions (Cohen d=0.64) and interventions ranging between 5-8 weeks were more effective [27]. Studies also suggest that internet-based interventions for stress can have sustained effects on stress reduction [31-33], be cost-effective [34,35], and have positive effects on participants' experiences of health and well-being in both work and private life [36]. However, previous internet-based cognitive behavioral therapy (iCBT) studies have focused on individuals with elevated stress, and few studies have evaluated the efficacy of iCBT in clinical samples such as employees with stress-related disorders. In addition, we found no previous studies evaluating the efficacy of internet-based and work-focused interventions for SA and RTW.

2b) In INTRODUCTION: Specific objectives or hypotheses

Does your paper address CONSORT subitem 2b? *

Copy and paste relevant sections from the manuscript (include quotes in quotation marks "like this" to indicate direct quotes from your manuscript), or elaborate on this item by providing additional information not in the ms, or briefly explain why the item is not applicable/relevant for your study

The aim of this study was to evaluate the efficacy of a work-focused iCBT (W-iCBT) intervention compared with generic iCBT and a waitlist control (WLC) group in a self-referred sample of employees with stress-related disorders. We hypothesized that W-iCBT and iCBT would be superior in reducing perceived stress, burnout, and exhaustion and improving recovery from work and quality of life compared with a WLC group. In secondary explorative analyses, we examined whether the W-iCBT group would differ from the iCBT and WLC groups in terms of important work-related outcomes, including work experience, work ability, SA, and long-term sick leave. We also hypothesized that the initially achieved changes would remain stable at the 12-month follow-up period.

METHODS

3a) Description of trial design (such as parallel, factorial) including allocation ratio

Does your paper address CONSORT subitem 3a? *

Copy and paste relevant sections from the manuscript (include quotes in quotation marks "like this" to indicate direct quotes from your manuscript), or elaborate on this item by providing additional information not in the ms, or briefly explain why the item is not applicable/relevant for your study

Design
This study was a 3-armed controlled superiority trial with 182 participants, and two internet-based interventions for stress-related disorders, namely, (1) iCBT (n=61, 33.5%) and (2) W-iCBT (n=61, 33.5%) that integrated work aspects early into the treatment, were compared against a WLC group (n=60, 33%).

3b) Important changes to methods after trial commencement (such as eligibility criteria), with reasons

Does your paper address CONSORT subitem 3b? *

Copy and paste relevant sections from the manuscript (include quotes in quotation marks "like this" to indicate direct quotes from your manuscript), or elaborate on this item by providing additional information not in the ms, or briefly explain why the item is not applicable/relevant for your study

Not applicable/relevant for our study.

3b-i) Bug fixes, Downtimes, Content Changes

Bug fixes, Downtimes, Content Changes: ehealth systems are often dynamic systems. A description of changes to methods therefore also includes important changes made on the intervention or comparator during the trial (e.g., major bug fixes or changes in the functionality or content) (5-iii) and other "unexpected events" that may have influenced study design such as staff changes, system failures/downtimes, etc. [2].

subitem not at all important

1 (selected)

2

3

4

5

essential

Clear selection

Does your paper address subitem 3b-i?

Copy and paste relevant sections from the manuscript (include quotes in quotation marks "like this" to indicate direct quotes from your manuscript), or elaborate on this item by providing additional information not in the ms, or briefly explain why the item is not applicable/relevant for your study

Not applicable/relevant for our study.

4a) Eligibility criteria for participants

Does your paper address CONSORT subitem 4a? *

Copy and paste relevant sections from the manuscript (include quotes in quotation marks "like this" to indicate direct quotes from your manuscript), or elaborate on this item by providing additional information not in the ms, or briefly explain why the item is not applicable/relevant for your study

Inclusion and Exclusion Criteria
The participants were employees who volunteered to participate in the trial. To be eligible for the study, they had to fulfill the criteria for an adjustment disorder described in the subdivision F43 Reaction to severe stress and adjustment disorders of the ICD-10 [38]. The diagnosis was established through telephone interviews using the Mini International Neuropsychiatric Interview [39], additional criteria from the ICD-10; [25], and national diagnostic guidelines regarding stress-related disorders [9].

In addition to an adjustment disorder, participants must (1) be aged ≥18 years; (2) have Swedish proficiency; (3) have access to a computer or tablet computer with internet access; (4) be currently employed; and (5) have scored ≥4.4 points on SMBQ, ≤34 points on the Montgomery-Åsberg Depression Scale–Self-rating version (MADRS-S), ≤21 points on the Insomnia Severity Index (ISI), and ≤14 points on the Alcohol Use Disorders Identification Test (AUDIT). Mild to moderate forms of DSM-5 axis-I diagnosis [40] were accepted as comorbid conditions, as long as these were considered secondary to the primary adjustment disorder. Participants on full- or part-time sick leave, for ≤1 year, were also included.

Participants were excluded from the study if they (1) were currently in treatment for a stress-related disorder; (2) were currently experiencing bipolar disorder, psychosis, posttraumatic stress disorder, eating disorder, substance abuse, severe forms of depression, anxiety disorder, or personality disorders; or (3) had suicidal ideation based on item 9 of the MADRS-S. Participants on medication (eg, antidepressants or sleep medication) were not excluded from the study but were requested to keep their medication constant during the study period. In total, 489 individuals were screened and 307 (62.8%) were excluded according to the inclusion and exclusion criteria specified above.

4a-i) Computer / Internet literacy

Computer / Internet literacy is often an implicit “de facto” eligibility criterion - this should be explicitly clarified.

subitem not at all important

1 ○

2 ○

3 ○

4 ○

5 ◉

essential

Clear selection

Does your paper address subitem 4a-i?

Copy and paste relevant sections from the manuscript (include quotes in quotation marks "like this" to indicate direct quotes from your manuscript), or elaborate on this item by providing additional information not in the ms, or briefly explain why the item is not applicable/relevant for your study

"In addition to an adjustment disorder, participants must (1) be aged ≥18 years; (2) have Swedish proficiency; (3) have access to a computer or tablet computer with internet access"

4a-ii) Open vs. closed, web-based vs. face-to-face assessments:

Open vs. closed, web-based vs. face-to-face assessments: Mention how participants were recruited (online vs. offline), e.g., from an open access website or from a clinic, and clarify if this was a purely web-based trial, or there were face-to-face components (as part of the intervention or for assessment), i.e., to what degree got the study team to know the participant. In online-only trials, clarify if participants were quasi-anonymous and whether having multiple identities was possible or whether technical or logistical measures (e.g., cookies, email confirmation, phone calls) were used to detect/prevent these.

subitem not at all important

1 ○

2 ○

3 ○

4 ○

5 ◉

essential

Clear selection

Does your paper address subitem 4a-ii? *

Copy and paste relevant sections from the manuscript (include quotes in quotation marks "like this" to indicate direct quotes from your manuscript), or elaborate on this item by providing additional information not in the ms, or briefly explain why the item is not applicable/relevant for your study

After initial registration using a personal email address, potential participants received an ID number and were asked to (1) provide written informed consent, (2) complete web-based screening questionnaires (Measures section), and (3) participate in a diagnostic interview via telephone. Diagnostic interviews were conducted by licensed psychologists or master's-level psychology students under supervision. The master's-level psychology students underwent diagnostic training, and all diagnostic interviews were reviewed by a licensed psychologist. Following the interviews, the included participants were randomized. Participants in the iCBT and W-iCBT groups received access to the programs immediately after randomization, and participants in the WLC group received access to the W-iCBT program after the 6-month follow-up period.

4a-iii) Information giving during recruitment

Information given during recruitment. Specify how participants were briefed for recruitment and in the informed consent procedures (e.g., publish the informed consent documentation as appendix, see also item X26), as this information may have an effect on user self-selection, user expectation and may also bias results.

subitem not at all important

1 ○

2 ○

3 ○

4 ○

5 ◉

essential

Clear selection

Does your paper address subitem 4a-iii?

Copy and paste relevant sections from the manuscript (include quotes in quotation marks "like this" to indicate direct quotes from your manuscript), or elaborate on this item by providing additional information not in the ms, or briefly explain why the item is not applicable/relevant for your study

Detailed information and application to the study were presented on the project’s home page. After initial registration using a personal email address, potential participants received an ID number and were asked to (1) provide written informed consent

4b) Settings and locations where the data were collected

Does your paper address CONSORT subitem 4b? *

Copy and paste relevant sections from the manuscript (include quotes in quotation marks "like this" to indicate direct quotes from your manuscript), or elaborate on this item by providing additional information not in the ms, or briefly explain why the item is not applicable/relevant for your study

The study was conducted in a university setting, with researchers and the treatment platform hosted by the university. Participants were recruited from the public through advertisements, articles in regional and national newspapers, and labor organization magazines. Detailed information and application to the study were presented on the project's home page.

4b-i) Report if outcomes were (self-)assessed through online questionnaires

Clearly report if outcomes were (self-)assessed through online questionnaires (as common in web-based trials) or otherwise.

subitem not at all important

1 ○

2 ○

3 ○

4 ○

5 ◉

essential

Clear selection

Does your paper address subitem 4b-i? *

Copy and paste relevant sections from the manuscript (include quotes in quotation marks "like this" to indicate direct quotes from your manuscript), or elaborate on this item by providing additional information not in the ms, or briefly explain why the item is not applicable/relevant for your study

Outcomes were self-assessed through online questionnaires, "After initial registration using a personal email address, potential participants received an ID number and were asked to (1) provide written informed consent, (2) complete web-based screening questionnaires (Measures section), and (3) participate in a diagnostic interview via telephone."

4b-ii) Report how institutional affiliations are displayed

Report how institutional affiliations are displayed to potential participants [on ehealth media], as affiliations with prestigious hospitals or universities may affect volunteer rates, use, and reactions with regards to an intervention.(Not a required item – describe only if this may bias results)

subitem not at all important

1 (selected)

2

3

4

5

essential

Clear selection

Does your paper address subitem 4b-ii?

Copy and paste relevant sections from the manuscript (include quotes in quotation marks "like this" to indicate direct quotes from your manuscript), or elaborate on this item by providing additional information not in the ms, or briefly explain why the item is not applicable/relevant for your study

Not applicable/relevant.

5) The interventions for each group with sufficient details to allow replication, including how and when they were actually administered

5-i) Mention names, credential, affiliations of the developers, sponsors, and owners

Mention names, credential, affiliations of the developers, sponsors, and owners [6] (if authors/evaluators are owners or developer of the software, this needs to be declared in a “Conflict of interest” section or mentioned elsewhere in the manuscript).

subitem not at all important

- 1 (selected)
- 2
- 3
- 4
- 5

essential

Clear selection

Does your paper address subitem 5-i?

Copy and paste relevant sections from the manuscript (include quotes in quotation marks "like this" to indicate direct quotes from your manuscript), or elaborate on this item by providing additional information not in the ms, or briefly explain why the item is not applicable/relevant for your study

No conflict of interest was declared.

5-ii) Describe the history/development process

Describe the history/development process of the application and previous formative evaluations (e.g., focus groups, usability testing), as these will have an impact on adoption/use rates and help with interpreting results.

subitem not at all important

1 (selected)

2

3

4

5

essential

Clear selection

Does your paper address subitem 5-ii?

Copy and paste relevant sections from the manuscript (include quotes in quotation marks "like this" to indicate direct quotes from your manuscript), or elaborate on this item by providing additional information not in the ms, or briefly explain why the item is not applicable/relevant for your study

The content of the programs was based on and previous studies, focus groups, usability testing.

5-iii) Revisions and updating

Revisions and updating. Clearly mention the date and/or version number of the application/intervention (and comparator, if applicable) evaluated, or describe whether the intervention underwent major changes during the evaluation process, or whether the development and/or content was “frozen” during the trial. Describe dynamic components such as news feeds or changing content which may have an impact on the replicability of the intervention (for unexpected events see item 3b).

subitem not at all important

- 1 (selected)
- 2
- 3
- 4
- 5

essential

Clear selection

Does your paper address subitem 5-iii?

Copy and paste relevant sections from the manuscript (include quotes in quotation marks "like this" to indicate direct quotes from your manuscript), or elaborate on this item by providing additional information not in the ms, or briefly explain why the item is not applicable/relevant for your study

Not applicable/relevant.

5-iv) Quality assurance methods

Provide information on quality assurance methods to ensure accuracy and quality of information provided [1], if applicable.

subitem not at all important

1 (selected)

2

3

4

5

essential

Clear selection

Does your paper address subitem 5-iv?

Copy and paste relevant sections from the manuscript (include quotes in quotation marks "like this" to indicate direct quotes from your manuscript), or elaborate on this item by providing additional information not in the ms, or briefly explain why the item is not applicable/relevant for your study

Not applicable/relevant.

5-v) Ensure replicability by publishing the source code, and/or providing screenshots/screen-capture video, and/or providing flowcharts of the algorithms used

Ensure replicability by publishing the source code, and/or providing screenshots/screen-capture video, and/or providing flowcharts of the algorithms used. Replicability (i.e., other researchers should in principle be able to replicate the study) is a hallmark of scientific reporting.

subitem not at all important

1 (selected)

2

3

4

5

essential

Clear selection

Does your paper address subitem 5-v?

Copy and paste relevant sections from the manuscript (include quotes in quotation marks "like this" to indicate direct quotes from your manuscript), or elaborate on this item by providing additional information not in the ms, or briefly explain why the item is not applicable/relevant for your study

Not applicable/relevant.

5-vi) Digital preservation

Digital preservation: Provide the URL of the application, but as the intervention is likely to change or disappear over the course of the years; also make sure the intervention is archived (Internet Archive, webcitation.org, and/or publishing the source code or screenshots/videos alongside the article). As pages behind login screens cannot be archived, consider creating demo pages which are accessible without login.

subitem not at all important

- (●) 1
- ( ) 2
- ( ) 3
- ( ) 4
- ( ) 5

essential

Clear selection

Does your paper address subitem 5-vi?

Copy and paste relevant sections from the manuscript (include quotes in quotation marks "like this" to indicate direct quotes from your manuscript), or elaborate on this item by providing additional information not in the ms, or briefly explain why the item is not applicable/relevant for your study

Not applicable/relevant.

5-vii) Access

Access: Describe how participants accessed the application, in what setting/context, if they had to pay (or were paid) or not, whether they had to be a member of specific group. If known, describe how participants obtained “access to the platform and Internet” [1]. To ensure access for editors/reviewers/readers, consider to provide a “backdoor” login account or demo mode for reviewers/readers to explore the application (also important for archiving purposes, see vi).

subitem not at all important

- 1 ○
- 2 ○
- 3 ◉
- 4 ○
- 5 ○

essential

Clear selection

Does your paper address subitem 5-vii? *

Copy and paste relevant sections from the manuscript (include quotes in quotation marks "like this" to indicate direct quotes from your manuscript), or elaborate on this item by providing additional information not in the ms, or briefly explain why the item is not applicable/relevant for your study

Participation was free of charge. Eligible participants obtained access to the platform via web/Internet and two factor authentication.

5-viii) Mode of delivery, features/functionalities/components of the intervention and comparator, and the theoretical framework

Describe mode of delivery, features/functionalities/components of the intervention and comparator, and the theoretical framework [6] used to design them (instructional strategy [1], behaviour change techniques, persuasive features, etc., see e.g., [7, 8] for terminology). This includes an in-depth description of the content (including where it is coming from and who developed it) [1],” whether [and how] it is tailored to individual circumstances and allows users to track their progress and receive feedback” [6]. This also includes a description of communication delivery channels and – if computer-mediated communication is a component – whether communication was synchronous or asynchronous [6]. It also includes information on presentation strategies [1], including page design principles, average amount of text on pages, presence of hyperlinks to other resources, etc. [1].

subitem not at all important

1 ○

2 ○

3 ◉

4 ○

5 ○

essential

Clear selection

Does your paper address subitem 5-viii? *

Copy and paste relevant sections from the manuscript (include quotes in quotation marks "like this" to indicate direct quotes from your manuscript), or elaborate on this item by providing additional information not in the ms, or briefly explain why the item is not applicable/relevant for your study

The generic iCBT program, represented in the iCBT and W-iCBT groups, was based on contemporary CBT techniques adapted for stress-related disorders and recovery from work training inspired by Hahn et al [41]. Both the iCBT and W-iCBT programs consisted of 10 modules distributed over 10 weeks, with modules lasting 60-120 minute per week (Table 1). The W-iCBT was integrated and distributed over each module and compared with generic iCBT, adding correspondingly 1 to 3 regular pages of text, worksheets, and homework assignments. Each module contained information, exercises, worksheets, images, examples, audio and video files, and homework exercises. All participants were requested to complete each module and homework assignment before they were able to continue. Delayed participants were able to catch up during the last module of the program. All participants had access to the treatment 1 year after the posttreatment assessment.

5-ix) Describe use parameters

Describe use parameters (e.g., intended “doses” and optimal timing for use). Clarify what instructions or recommendations were given to the user, e.g., regarding timing, frequency, heaviness of use, if any, or was the intervention used ad libitum.

subitem not at all important

1 ○

2 ○

3 ◉

4 ○

5 ○

essential

Clear selection

Does your paper address subitem 5-ix?

Copy and paste relevant sections from the manuscript (include quotes in quotation marks "like this" to indicate direct quotes from your manuscript), or elaborate on this item by providing additional information not in the ms, or briefly explain why the item is not applicable/relevant for your study

Both the iCBT and W-iCBT programs consisted of 10 modules distributed over 10 weeks, with modules lasting 60-120 minute per week (Table 1). All participants were requested to complete each module (one module per week) and homework assignment before they were able to continue.

5-x) Clarify the level of human involvement

Clarify the level of human involvement (care providers or health professionals, also technical assistance) in the e-intervention or as co-intervention (detail number and expertise of professionals involved, if any, as well as “type of assistance offered, the timing and frequency of the support, how it is initiated, and the medium by which the assistance is delivered”. It may be necessary to distinguish between the level of human involvement required for the trial, and the level of human involvement required for a routine application outside of a RCT setting (discuss under item 21 – generalizability).

subitem not at all important

1 ○

2 ○

3 ○

4 ○

5 ◉

essential

Clear selection

Does your paper address subitem 5-x?

Copy and paste relevant sections from the manuscript (include quotes in quotation marks "like this" to indicate direct quotes from your manuscript), or elaborate on this item by providing additional information not in the ms, or briefly explain why the item is not applicable/relevant for your study

Every week, participants in both interventions (W-iCBT and iCBT) received personalized written messages from a coach with feedback on the exercises. For the participants in the W-iCBT group, guidance was given on the CBT and work-focused modules simultaneously. The coaches, 8 in total, were master-level psychology students who were specifically trained to provide feedback according to a standardized manual. The feedback aimed to provide support and encouragement and to monitor homework assignments and adherence to the intervention. Treatment-as-usual was not only prohibited but also not encouraged during the trial. The coaches were requested to limit their support to 1 message and a maximum of 15 minutes of correspondence per week with 1 participant.

5-xi) Report any prompts/reminders used

Report any prompts/reminders used: Clarify if there were prompts (letters, emails, phone calls, SMS) to use the application, what triggered them, frequency etc. It may be necessary to distinguish between the level of prompts/reminders required for the trial, and the level of prompts/reminders for a routine application outside of a RCT setting (discuss under item 21 – generalizability).

subitem not at all important

1 ○

2 ○

3 ○

4 ○

5 ○

essential

Does your paper address subitem 5-xi? *

Copy and paste relevant sections from the manuscript (include quotes in quotation marks "like this" to indicate direct quotes from your manuscript), or elaborate on this item by providing additional information not in the ms, or briefly explain why the item is not applicable/relevant for your study

Prompts/reminders used, e.g. manual and automated emails and SMS triggered by non-response on weekly support, homework, exercises and/or questionnaires.

5-xii) Describe any co-interventions (incl. training/support)

Describe any co-interventions (incl. training/support): Clearly state any interventions that are provided in addition to the targeted eHealth intervention, as ehealth intervention may not be designed as stand-alone intervention. This includes training sessions and support [1]. It may be necessary to distinguish between the level of training required for the trial, and the level of training for a routine application outside of a RCT setting (discuss under item 21 – generalizability.

subitem not at all important

1 ○

2 ○

3 ○

4 ○

5 ●

essential

Clear selection

Does your paper address subitem 5-xii? *

Copy and paste relevant sections from the manuscript (include quotes in quotation marks "like this" to indicate direct quotes from your manuscript), or elaborate on this item by providing additional information not in the ms, or briefly explain why the item is not applicable/relevant for your study

In the present trial we evalated a co- (work-focused) interventioner. The work-focused (W-iCBT) aimed to facilitate return to work among those participants who were on sick leave and increase work functioning among those participants who were experiencing an adjustment disorder but not disabled from work. Work-focused CBT is built on the same conceptual framework as regular CBT. For example, CBT principles are used to change the appraisal of work stressors (eg, “it is ok although the task is not 100% complete or perfect”), change dysfunctional behavior (eg, working late close to bedtime and accepting more work despite heavy workload), or increase health-promoting behaviors (eg, recreational activities, assertive behavior, and RTW activities). The CBT principle of exposure has received special attention. Gradual exposure can help individuals develop more effective coping skills when dealing with work-related stressors (eg, assertiveness) and stimulate a gradual RTW setting for individuals on long-term sick leave [21].

6a) Completely defined pre-specified primary and secondary outcome measures, including how and when they were assessed

Does your paper address CONSORT subitem 6a? *

Copy and paste relevant sections from the manuscript (include quotes in quotation marks "like this" to indicate direct quotes from your manuscript), or elaborate on this item by providing additional information not in the ms, or briefly explain why the item is not applicable/relevant for your study

Primary and Secondary Outcome Measure was assessed pre, post and on a six and twelve month follow-up. Please see specifikation of the outcome measures below.

Primary Outcome Measure

Shirom Melamed Burnout Questionnaire
The Shirom Melamed Burnout Questionnaire, SMBQ [48,49] is a 22-item scale (graded 1-7) used to assess different aspects of chronic stress and burnout (physical fatigue, cognitive weariness, tension, and listlessness). This scale correlates significantly with other well-established questionnaires measuring burnout, eg, Maslach Burnout Inventory [49]. The SMBQ has exhibited good internal consistency with a Cronbach α of .92 [47] and in this study, indicated by an α of .84.

Secondary Outcome Measures

Perceived Stress Scale
Perceived stress was measured using the 10-item version of the Perceived Stress Scale (PSS-14), translated into Swedish [50,51]. The PSS-10 is designed to measure the degree to which situations in one's life are appraised as stressful. The Swedish version of the PSS has an internal consistency reliability (Cronbach α) of .82 and in the present sample of .77.
Karolinska Exhaustion Disorder Scale
Karolinska Exhaustion Disorder Scale (KEDS-9) is a 9-item questionnaire measuring symptoms of chronic stress, fatigue, and exhaustion [52]. The instrument is answered on a 7-point scale, with a scale range of 0 to 54. A cutoff score of 19 was shown to discriminate between healthy participants and patients with chronic stress and exhaustion [52]. The KEDS-9 has satisfactory reliability, with a Cronbach α of .94 [52] and correspondingly .74 in for this trial.
Montgomery Åsberg Depression Rating Scale
We used the Montgomery Åsberg Depression Rating Scale self-assessment, MADRS-S [53] to measure symptoms of depression. The MADRS-S consists of 9 items measuring different symptoms of depression, and each symptom is rated on a 6-point scale. The instrument has good reliability [54] indicated by a Cronbach α of .75 in this study sample. In a comparative study [55], the MADRS-S correlated highly (r=.87) with the Beck Depression Inventory [56], indicating acceptable convergent validity.
Generalized Anxiety Disorder Scale
The Generalized Anxiety Disorder 7-item Scale (GAD-7) is an instrument used to assess excessive worry and generalized anxiety disorder. The GAD-7 has good internal consistency reliability (α=.83), test-retest reliability (r=.83), as well as criterion, construct, factorial, and procedural validity [57]. Cronbach α of .79 was obtained in this study. A cutoff score of 10 has been suggested to discriminate between healthy participants and patients with generalized anxiety disorders.
Insomnia Severity Index
The Insomnia Severity Index ISI is a 7-item self-report questionnaire that measures individuals' perceptions of their insomnia and the severity of problems with delayed sleep onset, sleep maintenance, and early morning awakenings [58]. The ISI exhibits adequate internal consistency measures (α=.74) and is a sensitive measure for detecting changes in perceived sleep difficulties [58]. In this study sample, the Cronbach α was .86. It has previously been validated as an internet-based measure [59].
Alcohol Use Disorders Identification Test
The Alcohol Use Disorders Identification Test, AUDIT [60] was used to assess potential alcohol dependence or abuse. In a study of the psychometric properties of the Swedish version of the AUDIT, both internal and test-retest reliabilities were satisfactory [61]. In this

version of the AUDIT, both internal and test-retest reliabilities were satisfactory [61]. In this study, the Cronbach α was .65. A cutoff of <14 points on the AUDIT indicates a risk of alcohol overconsumption [62].
Work Experience Measurement Scale
The Work Experience Measurement Scale (WEMS) is an instrument measuring the experience of work from a health resource perspective [63]. The WEMS consists of 32 items that measure job satisfaction in 5 different domains (supportive work conditions, internal work experience, autonomy, time experience, management, and process of change) on a 6-point scale. Cronbach α for the WEMS was reported to be in the interval of .85 to .96 [63] and .94 in the present sample.
Work Ability Index
The Work Ability Index (WAI) is an instrument used to assess health status and work ability among employees [64-66]. The WAI comprises different scales, with scores ranging from 7 to 49. Studies [64] have suggested that 7 to 27 points indicate poor work ability; 28 to 36 points moderate ability; 37 to 43 points good ability; and 44 to 49 points indicate excellent work ability. Analyses of reliability indicate satisfactory internal consistency, with α levels ranging from .79 to .80 [67,68]. In this study, the α level was .66.
Sheehan Disability Scale
The Sheehan Disability Scale (SDS) measures quality of life and everyday function in 3 domains: work ability, social life, and family life (69;70). The instrument is answered on a 10-point visual analog scale, with a scale range of 0 to 30. The SDS has satisfactory internal consistency reliability (α=.89) and test-retest reliability (r=0.73; 42,43). α level was .66 in this study sample (69;70).
Recovery Experiences Questionnaire
The 16-item Recovery Experience Questionnaire (REQ) includes four factors, representing four different recovery experiences: (1) psychological detachment, (2) relaxation, (3) mastery, and (4) control [71]. The questionnaire is answered on a 5-point Likert scale and has been validated in a Swedish population, showing excellent internal consistency (α=.92) [72]. In this study, α level was .86.
Sickness Absence And Long-term Sick Leave
Absence from work was measured according to the Trimbos and Institute of Medical Technology Assessment Cost Questionnaire for Psychiatry (TiC-P) [73]. TiC-P has been used in several studies for economic evaluation of health care consumption and productivity loss in mental health [73]. Sickness absence (SA) was conceptualized as the self-rated number of days absent from work during the past 3 months while being physically or mentally ill. Long-term sick leave was operationalized as >15 days on sick leave and based on data from the Swedish Social Insurance Agency on the number of net days on sickness benefit between the pretreatment and 6-month follow-up assessments. In Sweden, sickness benefits from the Swedish Social Insurance Agency are due from day 15 on sick leave. Thus, absence during the first 14 days of illness was not included in the analysis of this outcome.
Intervention Utility And Satisfaction
The participants were asked to rate their utility and satisfaction after each module on a 5-point scale (1=low utility/satisfaction to 5=high utility/satisfaction).
Intervention Support
Intervention support was operationalized and assessed as the number of minutes of support per week between the coaches and the participants during the intervention.

6a-i) Online questionnaires: describe if they were validated for online use and apply CHERRIES items to describe how the questionnaires were designed/deployed

If outcomes were obtained through online questionnaires, describe if they were validated for online use and apply CHERRIES items to describe how the questionnaires were designed/deployed [9].

subitem not at all important

1 ○
2 ○
3 ○
4 ○
5 ◉

essential

Clear selection

Does your paper address subitem 6a-i?

Copy and paste relevant sections from manuscript text

Yes, they were validated for online use.

6a-ii) Describe whether and how “use” (including intensity of use/dosage) was defined/measured/monitored

Describe whether and how “use” (including intensity of use/dosage) was defined/measured/monitored (logins, logfile analysis, etc.). Use/adoption metrics are important process outcomes that should be reported in any ehealth trial.

subitem not at all important

1 ○

2 ○

3 ◉

4 ○

5 ○

essential

Clear selection

Does your paper address subitem 6a-ii?

Copy and paste relevant sections from manuscript text

On average, participants in the W-iCBT and iCBT groups completed 8.86 (SD 1.96) modules and 8.69 (SD 1.86) modules, respectively, which equals 88.6% and 86.9% of each intervention. completion was defined as the number of completed modules including all exercises and homework assignments. All exercises and homework assignments was logged in the platform. We also logged numer of logins and activities but this data was unreliable, incomplete and therefore unusable.

6a-iii) Describe whether, how, and when qualitative feedback from participants was obtained

Describe whether, how, and when qualitative feedback from participants was obtained (e.g., through emails, feedback forms, interviews, focus groups).

subitem not at all important

1 (selected)

2

3

4

5

essential

Clear selection

Does your paper address subitem 6a-iii?

Copy and paste relevant sections from manuscript text

Not applicable/relevant for our study.

6b) Any changes to trial outcomes after the trial commenced, with reasons

Does your paper address CONSORT subitem 6b? *

Copy and paste relevant sections from the manuscript (include quotes in quotation marks "like this" to indicate direct quotes from your manuscript), or elaborate on this item by providing additional information not in the ms, or briefly explain why the item is not applicable/relevant for your study

Not applicable/relevant for our study.

7a) How sample size was determined

NPT: When applicable, details of whether and how the clustering by care provides or centers was addressed

7a-i) Describe whether and how expected attrition was taken into account when calculating the sample size

Describe whether and how expected attrition was taken into account when calculating the sample size.

subitem not at all important

1 ○

2 ○

3 ◉

4 ○

5 ○

essential

Clear selection

Does your paper address subitem 7a-i?

Copy and paste relevant sections from manuscript title (include quotes in quotation marks "like this" to indicate direct quotes from your manuscript), or elaborate on this item by providing additional information not in the ms, or briefly explain why the item is not applicable/relevant for your study

Yes, expected attrition was taken into account when calculating the sample size.

"Estimates of sample size was based on previous controlled trials on iCBT for chronic stress [31], to detect an effect size of Cohen d=0.50 on the primary outcome of the Shirom-Melamed Burnout Questionnaire (SMBQ; Measures section) at posttreatment assessment, based on a power of 0.80 in a 2-tailed test with .05 significant level."

7b) When applicable, explanation of any interim analyses and stopping guidelines

Does your paper address CONSORT subitem 7b? *

Copy and paste relevant sections from the manuscript (include quotes in quotation marks "like this" to indicate direct quotes from your manuscript), or elaborate on this item by providing additional information not in the ms, or briefly explain why the item is not applicable/relevant for your study

Statistical Analysis
All analyses followed the CONSORT statement for randomized controlled trials [37]. Statistical analyses were conducted following the intention-to-treat (ITT) principle using SPSS (version 26; IBM Corp). We used the multiple imputation procedure to impute missing sum scores for participants who did not complete the posttreatment and 6- or 12-month follow-up assessments. Multiple imputation is considered a conservative approach for analyzing incomplete data sets, as it takes into account the uncertainty because of missing information [74]. We used all available data from the pretreatment, posttreatment, and 6-month follow-up assessments, as well as age, gender, andeducational level as predictors. Means, SDs, and SEs of the effect sizes were pooled from 5 sets of imputations. The effects of group on primary and secondary outcome measures of the ITT and completers-only data sets were analyzed using repeated measures ANOVAs with time (pretreatment, posttreatment, and 6-month follow-up period) as a within-subject factor. Pooled F values were calculated using RStudio (RStudio Inc). Cohen d was reported for between-group effect sizes and the corresponding 95% CI. Internal consistency reliability for the primary and secondary outcomes was analyzed using Cronbach α. Outcomes at baseline and demographic variables between complete and missing data were analyzed using t and χ2 tests. The ITT principle was applied to the analysis of SA. The analysis of long-term sick leave was based on complete registry data with no attrition. Both SA and long-term sick leave were analyzed using the Kruskal-Wallis nonparametric test, recommended for the comparison of ≥3 samples. To evaluate clinically significant changes, we used the guidelines by Jacobson and Truax [75]. Clinically significant changes were based on ITT analysis. To meet the criteria for clinically significant change in the primary outcome SMBQ, participants had to demonstrate a reliable change of 0.69 and score less than the cutoff of 4.4, following a recent study in a clinical sample [76]. We performed a clinically significant change analysis using the KEDS. On the KEDS, participants had to demonstrate a reliable change of 8.72 and score under the cutoff of 19 [52].

8a) Method used to generate the random allocation sequence

NPT: When applicable, how care providers were allocated to each trial group

Does your paper address CONSORT subitem 8a? *

Copy and paste relevant sections from the manuscript (include quotes in quotation marks "like this" to indicate direct quotes from your manuscript), or elaborate on this item by providing additional information not in the ms, or briefly explain why the item is not applicable/relevant for your study

Participants who met the study criteria and provided informed consent were randomly allocated by an independent researcher by using an internet-based random generator (Randomizer). The independent researcher received a list of anonymous identification numbers of all participants (n=182) and coaches (n=8). This procedure ensured that blinding was implemented during randomization. All participants, coaches, and the participants reporting benefits because of long-term sick leave at baseline were randomized in a 1:1:1 proportion.

8b) Type of randomisation; details of any restriction (such as blocking and block size)

Does your paper address CONSORT subitem 8b? *

Copy and paste relevant sections from the manuscript (include quotes in quotation marks "like this" to indicate direct quotes from your manuscript), or elaborate on this item by providing additional information not in the ms, or briefly explain why the item is not applicable/relevant for your study

Participants who met the study criteria and provided informed consent were randomly allocated by an independent researcher by using an internet-based random generator (Randomizer). The independent researcher received a list of anonymous identification numbers of all participants (n=182) and coaches (n=8). This procedure ensured that blinding was implemented during randomization. All participants, coaches, and the participants reporting benefits because of long-term sick leave at baseline were randomized in a 1:1:1 proportion.

9) Mechanism used to implement the random allocation sequence (such as sequentially numbered containers), describing any steps taken to conceal the sequence until interventions were assigned

Does your paper address CONSORT subitem 9? *

Copy and paste relevant sections from the manuscript (include quotes in quotation marks "like this" to indicate direct quotes from your manuscript), or elaborate on this item by providing additional information not in the ms, or briefly explain why the item is not applicable/relevant for your study

The independent researcher received a list of anonymous identification numbers of all participants (n=182) and coaches (n=8). This procedure ensured that blinding was implemented during randomization.

10) Who generated the random allocation sequence, who enrolled participants, and who assigned participants to interventions

Does your paper address CONSORT subitem 10? *

Copy and paste relevant sections from the manuscript (include quotes in quotation marks "like this" to indicate direct quotes from your manuscript), or elaborate on this item by providing additional information not in the ms, or briefly explain why the item is not applicable/relevant for your study

The independent researcher received a list of anonymous identification numbers of all participants (n=182) and coaches (n=8). This procedure ensured that blinding was implemented during randomization.

11a) If done, who was blinded after assignment to interventions (for example, participants, care providers, those assessing outcomes) and how

NPT: Whether or not administering co-interventions were blinded to group assignment

11a-i) Specify who was blinded, and who wasn’t

Specify who was blinded, and who wasn’t. Usually, in web-based trials it is not possible to blind the participants [1, 3] (this should be clearly acknowledged), but it may be possible to blind outcome assessors, those doing data analysis or those administering co-interventions (if any).

subitem not at all important

1 ○

2 ○

3 ○

4 ○

5 ◉

essential

Clear selection

Does your paper address subitem 11a-i? *

Copy and paste relevant sections from the manuscript (include quotes in quotation marks "like this" to indicate direct quotes from your manuscript), or elaborate on this item by providing additional information not in the ms, or briefly explain why the item is not applicable/relevant for your study

Only participants was blinded to the assigned to intervention. Providers was blinded to assessment but not to intervention.

11a-ii) Discuss e.g., whether participants knew which intervention was the “intervention of interest” and which one was the “comparator”

Informed consent procedures (4a-ii) can create biases and certain expectations - discuss e.g., whether participants knew which intervention was the “intervention of interest” and which one was the “comparator”.

subitem not at all important

1 ○

2 ○

3 ○

4 ○

5 ◉

essential

Clear selection

Does your paper address subitem 11a-ii?

Copy and paste relevant sections from the manuscript (include quotes in quotation marks "like this" to indicate direct quotes from your manuscript), or elaborate on this item by providing additional information not in the ms, or briefly explain why the item is not applicable/relevant for your study

Participants in the interventions group did not knew which intervention was the “intervention of interest”. Waitlist controls did know that they were comparators.

11b) If relevant, description of the similarity of interventions

(this item is usually not relevant for ehealth trials as it refers to similarity of a placebo or sham intervention to a active medication/intervention)

Does your paper address CONSORT subitem 11b? *

Copy and paste relevant sections from the manuscript (include quotes in quotation marks "like this" to indicate direct quotes from your manuscript), or elaborate on this item by providing additional information not in the ms, or briefly explain why the item is not applicable/relevant for your study

The generic iCBT and the work-focused iCBT were exact copies exept for the additional work-focused modules.

12a) Statistical methods used to compare groups for primary and secondary outcomes

NPT: When applicable, details of whether and how the clustering by care providers or centers was addressed

Does your paper address CONSORT subitem 12a? *

Copy and paste relevant sections from the manuscript (include quotes in quotation marks "like this" to indicate direct quotes from your manuscript), or elaborate on this item by providing additional information not in the ms, or briefly explain why the item is not applicable/relevant for your study

Statistical Analysis
All analyses followed the CONSORT statement for randomized controlled trials [37]. Statistical analyses were conducted following the intention-to-treat (ITT) principle using SPSS (version 26; IBM Corp). We used the multiple imputation procedure to impute missing sum scores for participants who did not complete the posttreatment and 6- or 12-month follow-up assessments. Multiple imputation is considered a conservative approach for analyzing incomplete data sets, as it takes into account the uncertainty because of missing information [74]. We used all available data from the pretreatment, posttreatment, and 6-month follow-up assessments, as well as age, gender, andeducational level as predictors. Means, SDs, and SEs of the effect sizes were pooled from 5 sets of imputations. The effects of group on primary and secondary outcome measures of the ITT and completers-only data sets were analyzed using repeated measures ANOVAs with time (pretreatment, posttreatment, and 6-month follow-up period) as a within-subject factor. Pooled F values were calculated using RStudio (RStudio Inc). Cohen d was reported for between-group effect sizes and the corresponding 95% CI. Internal consistency reliability for the primary and secondary outcomes was analyzed using Cronbach α. Outcomes at baseline and demographic variables between complete and missing data were analyzed using t and χ^2 tests. The ITT principle was applied to the analysis of SA. The analysis of long-term sick leave was based on complete registry data with no attrition. Both SA and long-term sick leave were analyzed using the Kruskal-Wallis nonparametric test, recommended for the comparison of ≥3 samples. To evaluate clinically significant changes, we used the guidelines by Jacobson and Truax [75]. Clinically significant changes were based on ITT analysis. To meet the criteria for clinically significant change in the primary outcome SMBQ, participants had to demonstrate a reliable change of 0.69 and score less than the cutoff of 4.4, following a recent study in a clinical sample [76]. We performed a clinically significant change analysis using the KEDS. On the KEDS, participants had to demonstrate a reliable change of 8.72 and score under the cutoff of 19 [52].

12a-i) Imputation techniques to deal with attrition / missing values

Imputation techniques to deal with attrition / missing values: Not all participants will use the intervention/comparator as intended and attrition is typically high in ehealth trials. Specify how participants who did not use the application or dropped out from the trial were treated in the statistical analysis (a complete case analysis is strongly discouraged, and simple imputation techniques such as LOCF may also be problematic [4]).

subitem not at all important

1 ○

2 ○

3 ○

4 ○

5 ◉

essential

Clear selection

Does your paper address subitem 12a-i? *

Copy and paste relevant sections from the manuscript (include quotes in quotation marks "like this" to indicate direct quotes from your manuscript), or elaborate on this item by providing additional information not in the ms, or briefly explain why the item is not applicable/relevant for your study

We used the multiple imputation procedure to impute missing sum scores for participants who did not complete the posttreatment and 6- or 12-month follow-up assessments. Multiple imputation is considered a conservative approach for analyzing incomplete data sets, as it takes into account the uncertainty because of missing information [74]. We used all available data from the pretreatment, posttreatment, and 6-month follow-up assessments, as well as age, gender, andeducational level as predictors. Means, SDs, and SEs of the effect sizes were pooled from 5 sets of imputations.

12b) Methods for additional analyses, such as subgroup analyses and adjusted analyses

Does your paper address CONSORT subitem 12b? *

Copy and paste relevant sections from the manuscript (include quotes in quotation marks "like this" to indicate direct quotes from your manuscript), or elaborate on this item by providing additional information not in the ms, or briefly explain why the item is not applicable/relevant for your study

Not applicable/relevant to our study.

X26) REB/IRB Approval and Ethical Considerations [recommended as subheading under "Methods"] (not a CONSORT item)

X26-i) Comment on ethics committee approval

subitem not at all important

1 ○

2 ○

3 ○

4 ○

5 ◉

essential

Clear selection

Does your paper address subitem X26-i?

Copy and paste relevant sections from the manuscript (include quotes in quotation marks "like this" to indicate direct quotes from your manuscript), or elaborate on this item by providing additional information not in the ms, or briefly explain why the item is not applicable/relevant for your study

Ethics Approval
The Ethical Committee of Linköping University, Sweden, approved all procedures involved in this study (reference number 2016/11-31). The study was registered retrospectively at ClinicalTrials.gov (NCT05240495).

x26-ii) Outline informed consent procedures

Outline informed consent procedures e.g., if consent was obtained offline or online (how? Checkbox, etc.?), and what information was provided (see 4a-ii). See [6] for some items to be included in informed consent documents.

subitem not at all important

1 ○

2 ○

3 ○

4 ○

5 ◉

essential

Clear selection

Does your paper address subitem X26-ii?

Copy and paste relevant sections from the manuscript (include quotes in quotation marks "like this" to indicate direct quotes from your manuscript), or elaborate on this item by providing additional information not in the ms, or briefly explain why the item is not applicable/relevant for your study

Informed consent was obtained on paper sent to each participant with returning mail.

X26-iii) Safety and security procedures

Safety and security procedures, incl. privacy considerations, and any steps taken to reduce the likelihood or detection of harm (e.g., education and training, availability of a hotline)

subitem not at all important

1 ○

2 ○

3 ○

4 ○

5 ◉

essential

Clear selection

Does your paper address subitem X26-iii?

Copy and paste relevant sections from the manuscript (include quotes in quotation marks "like this" to indicate direct quotes from your manuscript), or elaborate on this item by providing additional information not in the ms, or briefly explain why the item is not applicable/relevant for your study

All communication, assessment and requitement was conducted on an encrypted and two factor protected web platform, iTherapy. All participants received an anonymous ID number. ID and personal information were stored separately.

RESULTS

13a) For each group, the numbers of participants who were randomly assigned, received intended treatment, and were analysed for the primary outcome

NPT: The number of care providers or centers performing the intervention in each group and the number of patients treated by each care provider in each center

Does your paper address CONSORT subitem 13a? *

Copy and paste relevant sections from the manuscript (include quotes in quotation marks "like this" to indicate direct quotes from your manuscript), or elaborate on this item by providing additional information not in the ms, or briefly explain why the item is not applicable/relevant for your study

A total of 182 participants were randomized to the W-iCBT (61/182, 33.5%), iCBT (61/182, 33.5%), or WLC group (60/182, 33%).

13b) For each group, losses and exclusions after randomisation, together with reasons

Does your paper address CONSORT subitem 13b? (NOTE: Preferably, this is shown in a CONSORT flow diagram) *

Copy and paste relevant sections from the manuscript (include quotes in quotation marks "like this" to indicate direct quotes from your manuscript), or elaborate on this item by providing additional information not in the ms, or briefly explain why the item is not applicable/relevant for your study

After screening 489 individuals, 307 participants were excluded, most (n=197) because of high or low scores on one or several of the outcome measures.

Baseline data were available for all participants. Overall, the study attrition rate was moderate: 19.2% (35/182) at posttreatment period (W-iCBT, n=18, 51%; iCBT, n=9, 26%; and WLC, n=8, 23%), 24.2% (44/182) at the 6 months follow-up (W-iCBT, n=22, 50%; iCBT, n=11, 25%; and WLC, n=11, 25%), and 34.4% (42/122) at the 12-month follow-up questionnaires (W-iCBT=28 and iCBT=14). The analysis found no significant differences, χ23=.0645, n=182; P=.37, in the study attrition rate between any group or time point. No significant differences were found between demographic variables (presented in Table 2) or complete and missing data on the baseline outcome (Table 3).

13b-i) Attrition diagram

Strongly recommended: An attrition diagram (e.g., proportion of participants still logging in or using the intervention/comparator in each group plotted over time, similar to a survival curve) or other figures or tables demonstrating usage/dose/engagement.

subitem not at all important

1 ○

2 ○

3 ○

4 ○

5 ◉

essential

Clear selection

Does your paper address subitem 13b-i?

Copy and paste relevant sections from the manuscript or cite the figure number if applicable (include quotes in quotation marks "like this" to indicate direct quotes from your manuscript), or elaborate on this item by providing additional information not in the ms, or briefly explain why the item is not applicable/relevant for your study

Overall, the study attrition rate was moderate: 19.2% (35/182) at posttreatment period (W-iCBT, n=18, 51%; iCBT, n=9, 26%; and WLC, n=8, 23%), 24.2% (44/182) at the 6 months follow-up (W-iCBT, n=22, 50%; iCBT, n=11, 25%; and WLC, n=11, 25%), and 34.4% (42/122) at the 12-month follow-up questionnaires (W-iCBT=28 and iCBT=14). The analysis found no significant differences, χ23=.0645, n=182; P=.37, in the study attrition rate between any group or time point. No significant differences were found between demographic variables (presented in Table 2) or complete and missing data on the baseline outcome (Table 3).

14a) Dates defining the periods of recruitment and follow-up

Does your paper address CONSORT subitem 14a? *

Copy and paste relevant sections from the manuscript (include quotes in quotation marks "like this" to indicate direct quotes from your manuscript), or elaborate on this item by providing additional information not in the ms, or briefly explain why the item is not applicable/relevant for your study

No, we did not provide any dates.

14a-i) Indicate if critical “secular events” fell into the study period

Indicate if critical “secular events” fell into the study period, e.g., significant changes in Internet resources available or “changes in computer hardware or Internet delivery resources”

subitem not at all important

- 1 (selected)
- 2
- 3
- 4
- 5

essential

Clear selection

Does your paper address subitem 14a-i?

Copy and paste relevant sections from the manuscript (include quotes in quotation marks "like this" to indicate direct quotes from your manuscript), or elaborate on this item by providing additional information not in the ms, or briefly explain why the item is not applicable/relevant for your study

No, we avoided secular events, e.g. trial was conducted mid term, i.e. feb-may and sept-dec, not over longer vacations (summer/Christmas).

14b) Why the trial ended or was stopped (early)

Does your paper address CONSORT subitem 14b? *

Copy and paste relevant sections from the manuscript (include quotes in quotation marks "like this" to indicate direct quotes from your manuscript), or elaborate on this item by providing additional information not in the ms, or briefly explain why the item is not applicable/relevant for your study

Not applicable/relevant. The trial was not ended or stopped early.

15) A table showing baseline demographic and clinical characteristics for each group

NPT: When applicable, a description of care providers (case volume, qualification, expertise, etc.) and centers (volume) in each group

Does your paper address CONSORT subitem 15? *

Copy and paste relevant sections from the manuscript (include quotes in quotation marks "like this" to indicate direct quotes from your manuscript), or elaborate on this item by providing additional information not in the ms, or briefly explain why the item is not applicable/relevant for your study

Yes, we provided a table showing baseline demographic and clinical characteristics for each group.

15-i) Report demographics associated with digital divide issues

In ehealth trials it is particularly important to report demographics associated with digital divide issues, such as age, education, gender, social-economic status, computer/Internet/ehealth literacy of the participants, if known.

subitem not at all important

- 1 ○
- 2 ○
- 3 ○
- 4 ○
- 5 ◉

essential

Clear selection

Does your paper address subitem 15-i? *

Copy and paste relevant sections from the manuscript (include quotes in quotation marks "like this" to indicate direct quotes from your manuscript), or elaborate on this item by providing additional information not in the ms, or briefly explain why the item is not applicable/relevant for your study

Yes, we reported demographics associated with digital divide issues, age, education, gender, social-economic status.

16) For each group, number of participants (denominator) included in each analysis and whether the analysis was by original assigned groups

16-i) Report multiple “denominators” and provide definitions

Report multiple “denominators” and provide definitions: Report N’s (and effect sizes) “across a range of study participation [and use] thresholds” [1], e.g., N exposed, N consented, N used more than x times, N used more than y weeks, N participants “used” the intervention/comparator at specific pre-defined time points of interest (in absolute and relative numbers per group). Always clearly define “use” of the intervention.

subitem not at all important

1 ○

2 ○

3 ○

4 ○

5 ◉

essential

Clear selection

Does your paper address subitem 16-i? *

Copy and paste relevant sections from the manuscript (include quotes in quotation marks "like this" to indicate direct quotes from your manuscript), or elaborate on this item by providing additional information not in the ms, or briefly explain why the item is not applicable/relevant for your study

Yes, we reported multiple “denominators” and provide definitions. For example

16-ii) Primary analysis should be intent-to-treat

Primary analysis should be intent-to-treat, secondary analyses could include comparing only “users”, with the appropriate caveats that this is no longer a randomized sample (see 18-i).

subitem not at all important

1 ○

2 ○

3 ○

4 ○

5 ◉

essential

Clear selection

Does your paper address subitem 16-ii?

Copy and paste relevant sections from the manuscript (include quotes in quotation marks "like this" to indicate direct quotes from your manuscript), or elaborate on this item by providing additional information not in the ms, or briefly explain why the item is not applicable/relevant for your study

We provided both primary analysis on intent-to-treat sample and secondary analysis including completers only.

17a) For each primary and secondary outcome, results for each group, and the estimated effect size and its precision (such as 95% confidence interval)

Does your paper address CONSORT subitem 17a? *

Copy and paste relevant sections from the manuscript (include quotes in quotation marks "like this" to indicate direct quotes from your manuscript), or elaborate on this item by providing additional information not in the ms, or briefly explain why the item is not applicable/relevant for your study

Primary Outcome Analyses
The mean values) and SD for all groups for the primary outcomes are presented in Table 3. As depicted in Table 4, the repeated measures ANOVA for the primary outcome, the SMBQ, revealed a significant overall effect (F4,358=5.39; P<.001) between the interventions (W-iCBT and generic iCBT) and WLC. In the following separate ANOVA, both the W-iCBT and iCBT showed lower scores on the primary outcome SMBQ at posttest (T2; F2,179=14.9; P<.001) and at the 6 months follow-up (T3; F2,179=7.47; P<.01) than the WLC. Large effect sizes of Cohen d were observed at the posttest (W-iCBT, d=1.00; 95% CI 0.57-1.43 and iCBT, d=0.83; 95% CI 0.41-1.25) and at the 6 months follow-up (W-iCBT, d=0.74; 95% CI 0.30-1.18 and iCBT, d=0.74; 95% CI 0.35-1.13). The repeated measures ANOVA found no significant differences between the 2 interventions at any time point on the primary outcome (SMBQ T1-T3; F1,120=0.019; P=.99).

Secondary Outcome Analysis
The mean values (M) and SD for all groups of secondary outcomes are presented in Table 3. Table 4 presents the results of the ITT analyses of the secondary outcomes. The repeated measures ANOVA found significant overall effects in favor of the two intervention groups for all outcomes at T2 and T3, apart from work experience (F4,358=1.40; P=.24) and work ability (F4,358=1.18; P=.32). In the following analyses of simple effects, we found significant improvement in the W-iCBT group, when compared with the WLC group, on work ability (T2; F2,179=4.61; P=.03 and T3; F2,179=1.87; P=.18) and SA (T2; H(2)=−23.58; P=.01 and T3; H(2)=−18.44; P=.03). At the 6-month follow-up, SA was 324 days lower in the W-iCBT group (median 0.00; R=66; H(2)=−18.43; P=.03) than in the iCBT group, and 445 days (median 2.00; R=70; H(2)=−18.44; P=.03) lower than in the WLC group (median 3.00; R=77). However, no significant differences were found in the net days of long-term sick leave between any groups (H(2)=−0.82; P=.66). The total net days on benefits owing to long-term sick leave were 1932 days in the W-iCBT group, 2328 days in the iCBT group, and 2435 days in the WLC group. Accordingly, 14 participants in the W-iCBT group were on long-term sick leave at T2 (6 ended and 3 started) and 9 participants at T3 (5 ended and 0 started). Corresponding figures for the iCBT and WLC groups at T2 were 17 and 15 participants, respectively (iCBT, 4 ended and 4 started; WLC, 5 ended and 3 started), and 11 and 10 participants at T3 (iCBT, 8 ended and 2 started; WLC, 10 ended and 5 started).

17a-i) Presentation of process outcomes such as metrics of use and intensity of use

In addition to primary/secondary (clinical) outcomes, the presentation of process outcomes such as metrics of use and intensity of use (dose, exposure) and their operational definitions is critical. This does not only refer to metrics of attrition (13-b) (often a binary variable), but also to more continuous exposure metrics such as "average session length". These must be accompanied by a technical description how a metric like a "session" is defined (e.g., timeout after idle time) [1] (report under item 6a).

subitem not at all important

1 (selected)

2

3

4

5

essential

Clear selection

Does your paper address subitem 17a-i?

Copy and paste relevant sections from the manuscript (include quotes in quotation marks "like this" to indicate direct quotes from your manuscript), or elaborate on this item by providing additional information not in the ms, or briefly explain why the item is not applicable/relevant for your study

Not applicable/relevant for our study.

17b) For binary outcomes, presentation of both absolute and relative effect sizes is recommended

Does your paper address CONSORT subitem 17b? *

Copy and paste relevant sections from the manuscript (include quotes in quotation marks "like this" to indicate direct quotes from your manuscript), or elaborate on this item by providing additional information not in the ms, or briefly explain why the item is not applicable/relevant for your study

Not applicable/relevant for our study.

18) Results of any other analyses performed, including subgroup analyses and adjusted analyses, distinguishing pre-specified from exploratory

Does your paper address CONSORT subitem 18? *

Copy and paste relevant sections from the manuscript (include quotes in quotation marks "like this" to indicate direct quotes from your manuscript), or elaborate on this item by providing additional information not in the ms, or briefly explain why the item is not applicable/relevant for your study

Long-Term Follow-Up
The mean scores in the primary and secondary outcomes were maintained or continued to improve in both intervention groups at the 12-month follow-up. Significant differences between the iCBT and W-iCBT groups (Appendix 1) were only seen on the SMBQ subscale tension (F1,688=5.80; P=.02) and REQ subscale psychological detachment (F1,688=6.11; P=.01).

Intervention Support
Participants received an equal amount of time (minutes per week) for support (W-iCBT mean 12.11, SD 7.76; iCBT mean 12.92, SD 7.08; F1,120=0.356; P=.55). In addition, the participants were asked questions about how they perceived the support. Overall, 90% (55/61) in the W-iCBT group and 96% (59/182) in the iCBT group experienced the support as relevant and helpful.

Completers-Only Analyses
Completers-only analysis revealed significant (P<.001) and larger effects for the primary outcome (SMBQ) at postassessment time point (W-iCBT, d=1.31; 95% CI 0.86-1.77 and iCBT, d=1.13; 95% CI 0.71-1.55) and at the 6-month follow-up (W-iCBT, d=0.98; 95% CI 0.53-1.43 and iCBT, d=0.88; 95% CI 0.46-1.30) compared with the ITT-analyses. Significant differences and larger effect sizes were also observed in the secondary outcomes (data not shown).

Clinically Significant Change
The number of participants fulfilling the criteria for clinically significant change on the ITT data on the SMBQ at postassessment time point was 56% (34/61) in the W-iCBT group, 47% (29/61) in the iCBT group, and 21% (13/60) in the WLC group. At the 6 months follow-up, the proportion of clinically significant changes were W-CBT, 47% (29/61), iCBT, 48% (30/61), and WLC, 37% (22/60), respectively. On the KEDS, the proportions were 26% (W-iCBT, 16/61), 23% (iCBT, 14/61), and 6% (WLC, 4/60), at the 6 months follow-up, they were W-CBT, 34% (21/61); iCBT, 28% (17/61); and WLC, 11% (7/60).

18-i) Subgroup analysis of comparing only users

A subgroup analysis of comparing only users is not uncommon in ehealth trials, but if done, it must be stressed that this is a self-selected sample and no longer an unbiased sample from a randomized trial (see 16-iii).

subitem not at all important

1 (selected)

2

3

4

5

essential

Clear selection

Does your paper address subitem 18-i?

Copy and paste relevant sections from the manuscript (include quotes in quotation marks "like this" to indicate direct quotes from your manuscript), or elaborate on this item by providing additional information not in the ms, or briefly explain why the item is not applicable/relevant for your study

Not applicable/relevant for our study.

19) All important harms or unintended effects in each group

(for specific guidance see CONSORT for harms)

Does your paper address CONSORT subitem 19? *

Copy and paste relevant sections from the manuscript (include quotes in quotation marks "like this" to indicate direct quotes from your manuscript), or elaborate on this item by providing additional information not in the ms, or briefly explain why the item is not applicable/relevant for your study

Not applicable/relevant for our study.

19-i) Include privacy breaches, technical problems

Include privacy breaches, technical problems. This does not only include physical “harm” to participants, but also incidents such as perceived or real privacy breaches [1], technical problems, and other unexpected/unintended incidents. “Unintended effects” also includes unintended positive effects [2].

subitem not at all important

- 1 (selected)
- 2
- 3
- 4
- 5

essential

Clear selection

Does your paper address subitem 19-i?

Copy and paste relevant sections from the manuscript (include quotes in quotation marks "like this" to indicate direct quotes from your manuscript), or elaborate on this item by providing additional information not in the ms, or briefly explain why the item is not applicable/relevant for your study

Not applicable/relevant for our study.

19-ii) Include qualitative feedback from participants or observations from staff/researchers

Include qualitative feedback from participants or observations from staff/researchers, if available, on strengths and shortcomings of the application, especially if they point to unintended/unexpected effects or uses. This includes (if available) reasons for why people did or did not use the application as intended by the developers.

subitem not at all important

1 (selected)

2

3

4

5

essential

Clear selection

Does your paper address subitem 19-ii?

Copy and paste relevant sections from the manuscript (include quotes in quotation marks "like this" to indicate direct quotes from your manuscript), or elaborate on this item by providing additional information not in the ms, or briefly explain why the item is not applicable/relevant for your study

Not applicable/relevant for our study.

DISCUSSION

22) Interpretation consistent with results, balancing benefits and harms, and considering other relevant evidence

NPT: In addition, take into account the choice of the comparator, lack of or partial blinding, and unequal expertise of care providers or centers in each group

22-i) Restate study questions and summarize the answers suggested by the data, starting with primary outcomes and process outcomes (use)

Restate study questions and summarize the answers suggested by the data, starting with primary outcomes and process outcomes (use).

subitem not at all important

1 ○

2 ○

3 ○

4 ○

5 ◉

essential

Clear selection

Does your paper address subitem 22-i? *

Copy and paste relevant sections from the manuscript (include quotes in quotation marks "like this" to indicate direct quotes from your manuscript), or elaborate on this item by providing additional information not in the ms, or briefly explain why the item is not applicable/relevant for your study

To the best of our knowledge, this study is the first trial examining a work-focused intervention and a generic internet-based intervention in a clinical sample of employees with stress-related disorders. The results confirmed the primary hypothesis that both interventions were equally effective in reducing symptoms of perceived stress, burnout, exhaustion, depression, anxiety, and insomnia and in improving recovery from work and quality of life compared with a WLC group. Secondary explorative analyses indicated positive effects on work ability and a reduction in the number of days of SA in the work-focused group. No significant effects were found on outcomes for alcohol use, work experience, or net days on the benefits for long-term sick leave.

22-ii) Highlight unanswered new questions, suggest future research

Highlight unanswered new questions, suggest future research.

subitem not at all important

1 ○

2 ○

3 ○

4 ○

5 ◉

essential

Clear selection

Does your paper address subitem 22-ii?

Copy and paste relevant sections from the manuscript (include quotes in quotation marks "like this" to indicate direct quotes from your manuscript), or elaborate on this item by providing additional information not in the ms, or briefly explain why the item is not applicable/relevant for your study

The effects found on health-related outcomes were larger than those previously reported in a meta-analysis of internet-based stress management trials [27]; perceived stress d=0.43, depression d=0.34, and anxiety d=0.32. One plausible explanation may be that previous internet-based studies of interventions to reduce stress have largely included individuals with lower symptom severity (nonclinical). There are indications that greater initial symptom severity results in higher response and remission rates [77,78].
Most of the participants (W-iCBT, 42/61, 69%; iCBT, 41/61, 67%) in the intervention groups fulfilled the criteria for clinically significant change in the primary outcome, SMBQ, and were maintained at the 6-month follow-up. The proportion of clinically significant changes was comparable with previous trials [33,76]. However, there was a considerable discrepancy in the number of participants who achieved clinically significant changes when measured using KEDS compared with SMBQ. This might reflect that SMBQ and KEDS measure different underlying constructs, as noted in previous research [80].
As hypothesized, changes in health- and work-related outcomes remained stable in both intervention groups at the 12-month follow-up. The results were in line with the long-term effects found in a meta-analysis of internet-based stress management trials [27], which showed moderate effect sizes (d=0.56) up to 6 months after the treatment. These results are encouraging as they provide further evidence of the long-term benefits of relativity short iCBT interventions. However, we still struggle with the fact that about one-third of patients relapse or continue to experience residual symptoms several years after treatment for stress-related disorders [81]. Future studies should examine the use of minimally invasive long-term remote patient monitoring to further extend the long-term effects of iCBT stress interventions.
Interestingly, the effects on work ability and SA were only seen between the W-iCBT and WLC groups. However, these effects were small but comparable with those found in a meta-analysis of psychological interventions for individuals in SA because of common mental disorders [19]; Hedges g=0.22 for work-focused CBT interventions. These results are promising because SA has direct effects on people's well-being and leads to large costs for society [16]. Although effects on SA were present in the W-iCBT group, no significant effects were present in any group with regard to net days on the benefits of long-term sick leave. One possible explanation could be that the 2 outcomes were assessed differently. SA was conceptualized as the self-rated number of days absent from work during the past 3 months while being physically or mentally ill and measured at 3 time points: pretreatment, posttreatment, and 6-month follow-up. However, long-term sick leave was based on data from the Swedish Social Insurance Agency on the number of net days on sickness benefit between the pretreatment and 6-month follow-up assessments. In Sweden, sickness benefits from the Swedish Social Insurance Agency are due from day 15 on sick leave. Thus, absence during the first 14 days of illness was not included in the analysis of this outcome. Accordingly, SA and long-term sick leave were assessed differently, with different starting points, conceptualizations, and time intervals.
Inspired by the recovery from work training by Hahn et al [41,71], we included modules corresponding to the subdimensions of the REQ, namely, psychological detachment, relaxation, mastery, and control. The effect in this study was larger compared with previous internet-based stress management studies, including recovery techniques and the REQ [46,82,83]. Generally, recovery is a component of psychological treatment for stress and burnout. However, few studies have focused exclusively on recovery training. Consequently, it would be interesting to develop and evaluate an internet-based recovery training program, which exclusively focuses on various recovery skills. Hopefully, this can be an accessible and successful way to prevent stress-related problems in the working population.

20) Trial limitations, addressing sources of potential bias, imprecision, and, if relevant, multiplicity of analyses

20-i) Typical limitations in ehealth trials

Typical limitations in ehealth trials: Participants in ehealth trials are rarely blinded. Ehealth trials often look at a multiplicity of outcomes, increasing risk for a Type I error. Discuss biases due to non-use of the intervention/usability issues, biases through informed consent procedures, unexpected events.

subitem not at all important

1 ○

2 ○

3 ○

4 ○

5 ◉

essential

Clear selection

Does your paper address subitem 20-i? *

Copy and paste relevant sections from the manuscript (include quotes in quotation marks "like this" to indicate direct quotes from your manuscript), or elaborate on this item by providing additional information not in the ms, or briefly explain why the item is not applicable/relevant for your study

This study has several limitations. First, although this study focused on recruiting participants who were on sick leave because of stress and burnout, only 51 (28%) of 182 received sickness benefits at T1 and T3, resulting in unsatisfactory power in the statistical analysis. It is possible that the use of an open recruitment strategy and the fact that the intervention was delivered from an external institution (the university) may have had an impact on the recruitment. Future studies could use another recruitment and delivery approach, for example, directly via primary and occupational health care, to include more participants who are on sick leave because of stress-related illness. Second, by using an open recruitment strategy, we cannot rule out the risk of potential selection bias, selecting those cases that are most motivated to participate and willing to change. For example, 92% (167/182) of the participants had a university-level educational background, compared with 28% in the general population [84], and 62% (113/182) were working in the social, health care, or education sector. Therefore, future studies that include participants that are more representative of the general working population are needed. Providing W-iCBT directly, integrated into the workplace, could lower thresholds and be a successful approach in including various employees from different industries. Third, the overall study attrition rate was moderate; however, it was twice as large in W-iCBT (T2, 18/61, %; T3, 22/61, %) compared with generic iCBT (T2, 9/61, %; T3, 11 /61, %). Consistent with previous research [36], this might be due to the work-focused content (3 regular pages including text and corresponding worksheets and homework assignments) included in the W-iCBT and the extra workload relative to the generic iCBT intervention, constituting a potential stressor. Perhaps individualization and integration of the work-focused content could further streamline the treatment protocol, increase adherence, and prevent dropout. Fourth, we did not include any mediator or moderator analysis. Hence, future studies should be designed with repeated assessments to test for mediating and moderating mechanisms. For instance, it would be interesting to examine the mediation role of recovery in interventions for stress-related disorders. Fifth, none of the interventions examined in this trial included workplace involvement. Workplace dialogue and involvement (eg, manager, human resource professionals) were only encouraged indirectly through the participants. Workplace involvement is an important factor for returning to work [13,85]. Hence, it would be interesting to evaluate an internet-based and work-focused program for employees experiencing stress-related disorders with a parallel program including workplace involvement (eg, managerial support and perspective on stress, burnout, recovery, RTW, etc).

21) Generalisability (external validity, applicability) of the trial findings

NPT: External validity of the trial findings according to the intervention, comparators, patients, and care providers or centers involved in the trial

21-i) Generalizability to other populations

Generalizability to other populations: In particular, discuss generalizability to a general Internet population, outside of a RCT setting, and general patient population, including applicability of the study results for other organizations

subitem not at all important

1 ○

2 ○

3 ○

4 ○

5 ◉

essential

Clear selection

Does your paper address subitem 21-i?

Copy and paste relevant sections from the manuscript (include quotes in quotation marks "like this" to indicate direct quotes from your manuscript), or elaborate on this item by providing additional information not in the ms, or briefly explain why the item is not applicable/relevant for your study

With regard to the Generalizability:
92% (167/182) of the participants had a university-level educational background, compared with 28% in the general population [84], and 62% (113/182) were working in the social, health care, or education sector. Therefore, future studies that include participants that are more representative of the general working population are needed. Providing W-iCBT directly, integrated into the workplace, could lower thresholds and be a successful approach in including various employees from different industries.

None of the interventions examined in this trial included workplace involvement. Workplace dialogue and involvement (eg, manager, human resource professionals) were only encouraged indirectly through the participants. Workplace involvement is an important factor for returning to work [13,85]. Hence, it would be interesting to evaluate an internet-based and work-focused program for employees experiencing stress-related disorders with a parallel program including workplace involvement (eg, managerial support and perspective on stress, burnout, recovery, RTW, etc).

21-ii) Discuss if there were elements in the RCT that would be different in a routine application setting

Discuss if there were elements in the RCT that would be different in a routine application setting (e.g., prompts/reminders, more human involvement, training sessions or other co-interventions) and what impact the omission of these elements could have on use, adoption, or outcomes if the intervention is applied outside of a RCT setting.

subitem not at all important

1 (selected)

2

3

4

5

essential

Clear selection

Does your paper address subitem 21-ii?

Copy and paste relevant sections from the manuscript (include quotes in quotation marks "like this" to indicate direct quotes from your manuscript), or elaborate on this item by providing additional information not in the ms, or briefly explain why the item is not applicable/relevant for your study

Not applicable/relevant for our study.

OTHER INFORMATION

23) Registration number and name of trial registry

Does your paper address CONSORT subitem 23? *

Copy and paste relevant sections from the manuscript (include quotes in quotation marks "like this" to indicate direct quotes from your manuscript), or elaborate on this item by providing additional information not in the ms, or briefly explain why the item is not applicable/relevant for your study

The study was registered retrospectively at clinical trial: ClinicalTrials.gov, reference number NCT05240495.

24) Where the full trial protocol can be accessed, if available

Does your paper address CONSORT subitem 24? *

Cite a Multimedia Appendix, other reference, or copy and paste relevant sections from the manuscript (include quotes in quotation marks "like this" to indicate direct quotes from your manuscript), or elaborate on this item by providing additional information not in the ms, or briefly explain why the item is not applicable/relevant for your study

(J Med Internet Res 2023;25:e34446) doi: 10.2196/34446

25) Sources of funding and other support (such as supply of drugs), role of funders

Does your paper address CONSORT subitem 25? *

Copy and paste relevant sections from the manuscript (include quotes in quotation marks "like this" to indicate direct quotes from your manuscript), or elaborate on this item by providing additional information not in the ms, or briefly explain why the item is not applicable/relevant for your study

This study was sponsored by grants to the last author from the Swedish Council for Working and Life Research (FORTE) and Sweden's Municipalities and County Councils (SKL).

X27) Conflicts of Interest (not a CONSORT item)

X27-i) State the relation of the study team towards the system being evaluated

In addition to the usual declaration of interests (financial or otherwise), also state the relation of the study team towards the system being evaluated, i.e., state if the authors/evaluators are distinct from or identical with the developers/sponsors of the intervention.

subitem not at all important

1 ○

2 ○

3 ○

4 ○

5 ◉

essential

Clear selection

Does your paper address subitem X27-i?

Copy and paste relevant sections from the manuscript (include quotes in quotation marks "like this" to indicate direct quotes from your manuscript), or elaborate on this item by providing additional information not in the ms, or briefly explain why the item is not applicable/relevant for your study

Conflicts of Interest
None declared.

About the CONSORT EHEALTH checklist

As a result of using this checklist, did you make changes in your manuscript? *

○ yes, major changes

○ yes, minor changes

◉ no

What were the most important changes you made as a result of using this checklist?

Not applicable for our study.

How much time did you spend on going through the checklist INCLUDING making changes in your manuscript *

Not applicable for our study.

As a result of using this checklist, do you think your manuscript has improved? *

- ○ yes
- ◉ no
- ○ Other:

Would you like to become involved in the CONSORT EHEALTH group?

This would involve for example becoming involved in participating in a workshop and writing an "Explanation and Elaboration" document

- ○ yes
- ◉ no
- ○ Other:

Clear selection

Any other comments or questions on CONSORT EHEALTH

The CONSORT EHEALTH is very extensive (100+ pages) and should be properly condensed.

STOP - Save this form as PDF before you click submit

To generate a record that you filled in this form, we recommend to generate a PDF of this page (on a Mac, simply select "print" and then select "print as PDF") before you submit it.

When you submit your (revised) paper to JMIR, please upload the PDF as supplementary file.

Don't worry if some text in the textboxes is cut off, as we still have the complete information in our database. Thank you!

Final step: Click submit !

Click submit so we have your answers in our database!

Submit

Clear form

Never submit passwords through Google Forms.

This form was created outside of your domain. Report Abuse - Terms of Service - Privacy Policy

Google Forms
